# Towards reliable morphological subtyping and cancer detection on Barrett’s esophagus pathology through ensembling foundation models

**DOI:** 10.1038/s41746-026-03329-3

**Published:** 2026-10-21

**Authors:** Azar Kazemi, Camillo Saueressig, Jingsong Liu, Han Li, Julius Shakhtour, Bianca Grosser, Fabian Stögbauer, Julia Horstmann, Nassir Navab, Saeid Eslami, Michael Quante, Julia Slotta-Huspenina, Peter J. Schüffler

**Affiliations:** 1 Institute of Pathology, School of Medicine and Health, Technical University of Munich, Munich, Germany; 2 Department of Medical Informatics, School of Medicine, Mashhad University of Medical Sciences, Mashhad, Iran; 3 Munich Center for Machine Learning, Munich, Germany; 4 TUM School of Computation, Information and Technology, Technical University of Munich, Munich, Germany; 5 AI for Image-Guided Diagnosis and Therapy, Technical University of Munich, Munich, Germany; 6 Computer Aided Medical Procedures, Technical University of Munich, Munich, Germany; 7 Klinikum Rechts Der Isar, Medical Clinic and Polyclinic II, Technical University of Munich, Munich, Germany; 8 Pharmaceutical Sciences Research Center, Institute of Pharmaceutical Technology, Mashhad University of Medical Sciences, Mashhad, Iran; 9 Department of Medical Informatics, University of Amsterdam, Amsterdam, the Netherlands; 10 Department of Medicine II, University Medical Center Freiburg, Freiburg, Germany; 11 Department of Nephrology, School of Medicine, Technical University of Munich, Munich, Germany; 12 Pathologie Starnberg MVZ GmbH, Starnberg, Bayern, Germany; 13 Munich Data Science Institute, Munich, Germany

**Keywords:** Cancer, Computational biology and bioinformatics, Gastroenterology, Oncology

## Abstract

The reliability of emergent histopathology foundation models (Histo-FMs) for real-world diagnostic tasks remains unproven. Given the complexity of diagnostic tasks and the challenges inherent in real-world data, we utilized Histo-FMs to investigate their utility for morphological subtyping of Barrett’s esophagus (BE) and detecting esophageal adenocarcinoma (EAC) across the BE progression spectrum. We benchmarked Histo-FMs on the BarrettNet cohort, spanning diagnostic categories ranging from normal to EAC (N2EAC). The dataset comprised 3528 hematoxylin and eosin (H&E)-stained whole-slide images (WSIs) from 790 patients. A multi-magnification, multi-backbone aggregation of the five most expert-consistent single-scale models yielded incremental performance gains (AUROC of 0.907, F1-score of 0.696, accuracy of 0.795, and κ of 0.651 for morphological subtyping; AUROC of 0.909, F1-score of 0.836, accuracy of 0.959, and κ of 0.673 for EAC detection), with non-uniform gains across single-model comparisons and limited benefit for dysplasia. The findings support the capability of Histo-FMs for BE morphological subtyping and EAC detection in a multicenter clinical cohort, while highlighting persistent challenges in dysplasia assessment and the need for external validation. This study provides a foundation for future clinical validation.

## Introduction

Computational pathology has recently been revolutionized by self-supervised learning (SSL), leveraging massive, diverse, and unlabeled whole slide image (WSI) datasets to elevate model performance in pathology to a new level^1^. Novel histology foundation models (Histo-FMs) generate computational tissue representations using vision transformer (ViT) architectures^2^ that are transferable and generalizable across many histopathological tasks. Recent studies show that these Histo-FM embeddings perform well on public datasets^3,4^.

However, the reliability of Histo-FMs across a clinically heterogeneous diagnostic spectrum, i.e., the ability to produce consistent and accurate results beyond restricted diagnostic spectra and controlled research settings^5–7^, remains largely unknown. Benchmarking and validating the performance and consistency of these models on more realistic, inclusive diagnostic-cohort data, rather than on well-curated research datasets, is required. To further assess their clinical relevance and trustworthiness beyond conventional statistical performance metrics, it is crucial to evaluate the Histo-FMs’ concordance with pathologists. The reliability of Histo-FMs has recently come into focus^8^, but several dimensions, such as pairwise and inter-model agreement among Histo-FMs for a spectrum-wide diagnostic task, remain underexplored. Therefore, further research is needed to investigate this in greater depth.

Esophageal cancer is a common cancer with an incidence of more than 600 K cases worldwide^9^. Among all subtypes, esophageal adenocarcinoma (EAC) is growing most rapidly in the Western world^10^. Five-year survival rates range from 5% to 47%, depending on disease stage^11^. Given these dismal statistics, there is a great need to detect pre-cancerous stages before their progression to EAC. A well-known precursor of EAC is Barrett’s esophagus (BE), an inflammation-dependent condition of the esophagus characterized by a metaplastic change from squamous to columnar intestinal-type epithelium. Intestinal metaplasia can represent the initial step of esophageal carcinogenesis, potentially progressing from low-grade dysplasia (LGD) to high-grade dysplasia (HGD) and EAC^11^. As the prevalence of BE has increased tremendously over the last several decades, many individuals are “at risk” for this lethal malignancy^12^.

Significant resources are required for BE surveillance, aiming for early detection of HGD or EAC^13^. Previous studies have shown that EAC patients previously diagnosed with BE have a better survival^14^. Although non-invasive screening tools have emerged recently, endoscopy remains the most common screening approach worldwide^15,16^. Endoscopic surveillance can detect lesions that are biopsied and require histopathologic evaluation to render a final diagnosis^17–19^. Thus, to improve patient outcomes, provide timely and curative treatment, and reduce healthcare costs, automatic classification of BE states and the early detection of EAC are urgently required^16,17,20–25^. Histopathological assessment of Barrett-associated dysplasia remains challenging due to substantial interobserver variability, particularly in distinguishing reactive atypia, low-grade dysplasia, and high-grade dysplasia. This diagnostic uncertainty is clinically relevant, as dysplasia grading directly influences surveillance intervals, expert review, and treatment decisions.

The BarrettNET study^12,19^, a German multicenter cohort of BE patients was prospectively recruited with sample collection across 21 centers and a 10-year follow-up. This cohort comprises patients with an endoscopic diagnosis of BE (without dysplasia or EAC). During each patient’s visit, 3–6 standardized biopsy samples were collected from BE lesions or associated advanced lesions (dysplastic areas, EAC), as well as from tissue proximal and distal to the BE segment (squamous epithelium and cardia). Biopsies were fixed in PAXgene Tissue Fixative (Qiagen, Hilden, Germany) and sent to the Institute of Pathology at the Technical University of Munich for processing, paraffin embedding, and histopathological evaluation. Histopathological diagnoses were established and reviewed by expert gastrointestinal pathologists and served as the diagnostic reference standard for the cohort^12,19^. All procedures were conducted within the steps of an accredited diagnostic workflow (DIN EN ISO/IEC 17020).

Although formalin-fixed, paraffin-embedded tissue (FFPE) is the diagnostic gold standard, alternative fixation methods such as PAXgene-fixed, paraffin-embedded (PFPE) tissue have been proposed to improve biomolecular preservation while maintaining comparable histomorphological quality^26^. Previous ring-trial studies have further demonstrated the suitability of PFPE tissue for routine pathological assessment^26,27^. In addition, an international ring-trial study assessed histological evaluation of PFPE and FFPE in the BarrettNet cohort and demonstrated comparable diagnostic assessment and interobserver agreement among nine expert gastrointestinal pathologists^26^. Although the use of PFPE tissue did not reduce interobserver variability in dysplasia grading, the persistent variability highlights the inherent diagnostic challenges of Barrett-associated dysplasia^26^. The simultaneous preservation of morphology and biomolecules provides a valuable basis for integrated histopathological, molecular, and AI-enabled analyses within the BarrettNet translational research framework.

Deep learning is a promising tool for analyzing biopsy samples^28,29^. Recently, a data-efficient clustering-constrained multiple instance learning (MIL) called CLAM^30^ was proposed, which can offer strong slide-level classification across multiple tissue types^28,31,32^, particularly when a small dataset using only slide-level annotations is available^33,34^. CLAM^30^ was developed to reduce dependence on labor-intensive patch-level annotation in supervised learning^35^ and on the suboptimal localization of diagnostically informative regions within heterogeneous WSIs in classical weakly supervised learning. Therefore, this weakly supervised learning framework can be particularly advantageous for slide-level prediction of BE morphological subtyping within the progression spectrum.

Recent computational pathology research has focused on BE detection^16,36^, dysplasia grading^37^, normal-to-dysplastic tissue classification^38^, and dysplastic vs. non-dysplastic BE classification^39^. However, previous studies that encompass the full spectrum of BE progression, from normal tissue and Barrett’s metaplasia without dysplasia to LGD, HGD, and EAC, are rare^40^ and aim to closely reflect diagnostic complexity^41^. Incomplete clinical representation may lead to restricted diagnostic coverage and limit the utility of models for future diagnostic support in clinical practice. Moreover, diagnostic tasks require pattern recognition across all lesion categories; hence, the absence of progression stages may limit the translational potential of the findings. Incorporating a full spectrum of BE can address the limitations of prior BE studies, enhance diagnostic representativeness, and better capture morphologic variability. Therefore, inclusion of the pathological spectrum in progression-aware computational pathology can account for diagnostically ambiguous transition states and capture the biologically and diagnostically continuous nature of BE progression. This is particularly relevant for dysplastic lesions, which represent critical clinical decisions for surveillance, expert consultation, and therapeutic intervention.

The current study represents a comprehensive benchmarking of Histo-FMs for pathological diagnostic tasks in a full-spectrum diagnostic scenario. To address restricted diagnostic-spectrum coverage, we include a full diagnostic spectrum, from normal to EAC (N2EAC), for morphological subtyping of BE and EAC detection, and evaluate the effectiveness of the Histo-FMs within a weakly supervised learning framework (N2EAC-MIL) using a multicenter clinical cohort from a nationwide clinical effort. Despite extensive work on benchmarking Histo-FMs utilizing H&E-stained WSIs, their use on PFPE remains an open question, and their behavior on PFPE WSIs has yet to be explored. Therefore, we aim to evaluate the overall predictive performance of the nineteen Histo-FMs for clinically relevant diagnostic tasks across the BE progression spectrum using PFPE biopsy WSIs, specifically examining their behavior and interpretability for morphological subtyping and EAC detection.

## Results

### Dataset

The final curated dataset consisted of H&E-stained WSIs from 3528 histopathological slides of 790 patients. WSIs’ information was extracted and queried from the cohort dataset and reports obtained from the pathology information system, resulting in a dataset encompassing 1455 (41.24%) normal/non-Barrett’s tissues, 1697 (48.10%) Barrett’s without dysplasia, 145 (4.11%) Barrett’s with dysplasia, and 231 (6.55%) EAC. Dysplastic BE represented the smallest diagnostic class, reflecting the class imbalance inherent to real-world BE surveillance cohorts.

### Morphological subtyping

**Fig. 1:**
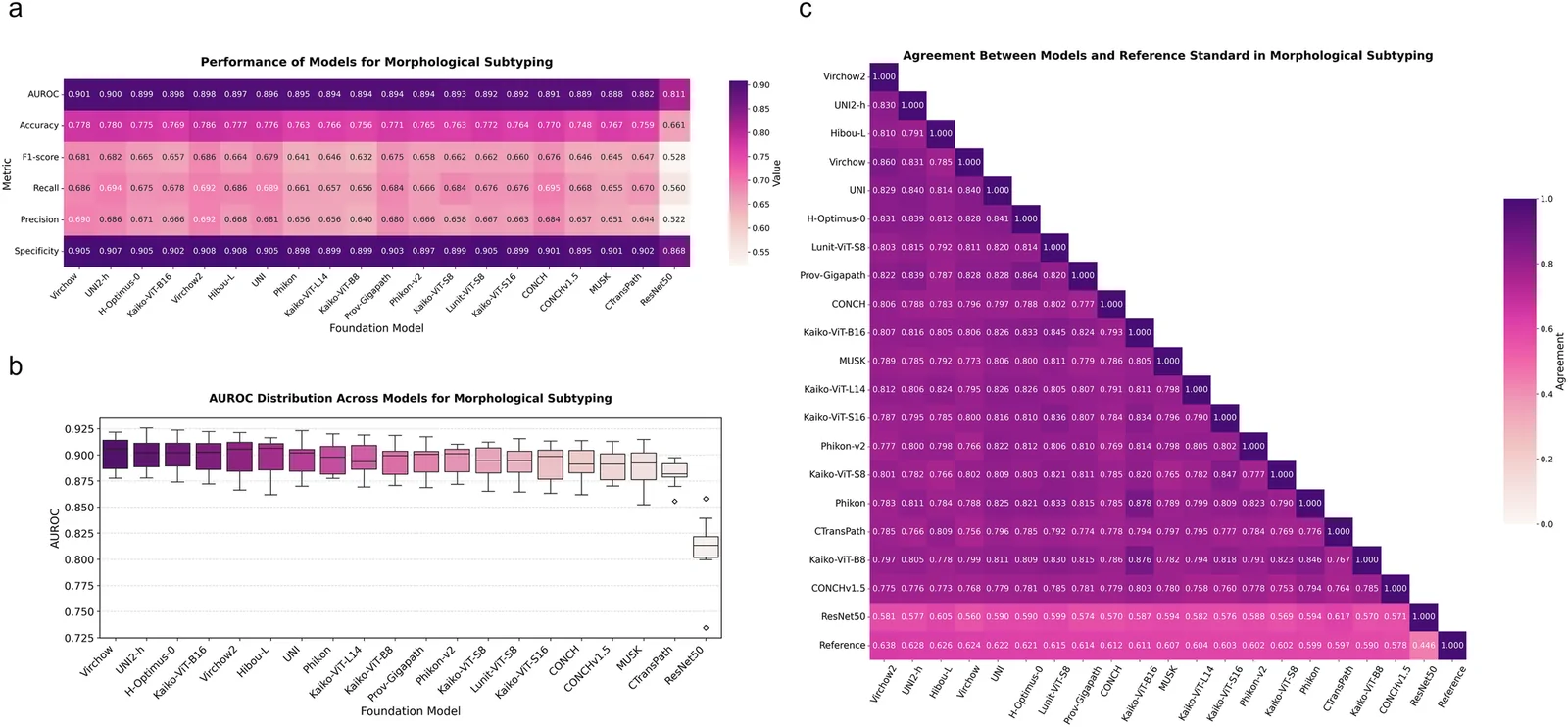
Models’ performance evaluation for morphological subtyping. **a** Heatmap illustrating N2EAC-MIL models’ performance for morphological subtyping. Models with the backbone of Virchow2, Virchow, UNI2-h, H-Optimus-0, and Kaiko-ViT-B16 achieved slightly higher AUROC scores than other models; **b** Each box plot summarizes the distribution of mean AUROC across all folds; **c** Pairwise agreement between N2EAC-MIL models and the reference standard in morphological subtyping. Virchow2, UNI2-h, Hibou-L, Virchow, and UNI achieved the highest agreement with the reference standard.

Figure 1 illustrates performance metrics across all N2EAC-MIL models integrated with different foundation models (FMs), including Histo-FMs and ResNet50. Performance metrics across a 10-fold cross validation revealed that morphological subtyping of BE using the Virchow model achieved the highest AUROC (mean ± standard deviation (SD): 0.901 ± 0.017 [95% CI ± 0.012]), while the Virchow2 model achieved AUROC of 0.898 ± 0.020 [95% CI ± 0.014], the highest F1-score (0.686 ± 0.023 [95% CI ± 0.016]). ResNet50 performed comparatively lower than any Histo-FM (Fig. 1a; Table 1). The results indicated that implementing the model with Virchow, UNI2-h, or H-Optimus-0 backbones yielded higher mean AUROC scores, although in some models the wider performance dispersion suggested less stability across folds (Fig. 1b). However, the CONCH model achieved a higher detection sensitivity for dysplasia (recall = 0.627). When we used Prov-Gigapath as the backbone of the N2EAC-MIL, the model achieved a higher EAC class recall (0.669) than other FMs (Table S1; Figs. S1–S2).

**Table 1:** Performance metrics for morphological subtyping

Foundation Model	Magnification	AUROC Mean ± SD [95% CI HW]	F1-Score Mean ± SD [95% CI HW]	Accuracy Mean ± SD [95% CI HW]	Precision Mean ± SD [95% CI HW]	Recall Mean ± SD [95% CI HW]	Specificity Mean ± SD [95% CI HW]	Cohen’s Kappa κ ± SD [95% CI HW]
Virchow2	20×	0.898 ± 0.020 [ ± 0.014]	0.686 ± 0.023 [ ± 0.016]	0.786 ± 0.012 [ ± 0.009]	0.692 ± 0.019 [ ± 0.014]	0.692 ± 0.036 [ ± 0.026]	0.908 ± 0.005 [ ± 0.004]	0.638 ± 0.017 [ ± 0.012]
Virchow	20×	0.901 ± 0.017 [ ± 0.012]	0.681 ± 0.049 [ ± 0.035]	0.778 ± 0.022 [ ± 0.016]	0.690 ± 0.049 [ ± 0.035]	0.686 ± 0.058 [ ± 0.041]	0.905 ± 0.008 [ ± 0.006]	0.624 ± 0.034 [ ± 0.024]
UNI2-h	20×	0.900 ± 0.016 [ ± 0.012]	0.682 ± 0.040 [ ± 0.029]	0.780 ± 0.016 [ ± 0.011]	0.686 ± 0.040 [ ± 0.029]	0.694 ± 0.057 [ ± 0.041]	0.907 ± 0.008 [ ± 0.006]	0.628 ± 0.023 [ ± 0.016]
UNI	20×	0.896 ± 0.017 [ ± 0.012]	0.679 ± 0.043 [ ± 0.031]	0.776 ± 0.019 [ ± 0.013]	0.681 ± 0.036 [ ± 0.026]	0.689 ± 0.062 [ ± 0.044]	0.905 ± 0.008 [ ± 0.006]	0.622 ± 0.028 [ ± 0.020]
ResNet50	20×	0.811 ± 0.032 [ ± 0.023]	0.528 ± 0.037 [ ± 0.026]	0.661 ± 0.023 [ ± 0.016]	0.522 ± 0.040 [ ± 0.028]	0.560 ± 0.041 [ ± 0.030]	0.868 ± 0.008 [ ± 0.006]	0.446 ± 0.034 [ ± 0.024]
Phikon-v2	20×	0.894 ± 0.015 [ ± 0.010]	0.658 ± 0.049 [ ± 0.035]	0.765 ± 0.018 [ ± 0.013]	0.666 ± 0.044 [ ± 0.032]	0.666 ± 0.063 [ ± 0.045]	0.897 ± 0.010 [ ± 0.007]	0.602 ± 0.025 [ ± 0.018]
Phikon	20×	0.895 ± 0.015 [ ± 0.011]	0.641 ± 0.050 [ ± 0.036]	0.763 ± 0.021 [ ± 0.015]	0.656 ± 0.034 [ ± 0.025]	0.661 ± 0.077 [ ± 0.055]	0.898 ± 0.013 [ ± 0.009]	0.599 ± 0.032 [ ± 0.023]
MUSK	20×	0.888 ± 0.020 [ ± 0.014]	0.645 ± 0.035 [ ± 0.025]	0.767 ± 0.018 [ ± 0.013]	0.651 ± 0.035 [ ± 0.025]	0.655 ± 0.045 [ ± 0.032]	0.901 ± 0.008 [ ± 0.006]	0.607 ± 0.025 [ ± 0.018]
Lunit-ViT-S8	20×	0.892 ± 0.016 [ ± 0.012]	0.662 ± 0.041 [ ± 0.030]	0.772 ± 0.026 [ ± 0.018]	0.667 ± 0.041 [ ± 0.029]	0.676 ± 0.057 [ ± 0.041]	0.905 ± 0.010 [ ± 0.007]	0.615 ± 0.038 [ ± 0.027]
Kaiko- ViT-S8	20×	0.893 ± 0.016 [ ± 0.011]	0.662 ± 0.052 [ ± 0.037]	0.763 ± 0.020 [ ± 0.015]	0.658 ± 0.046 [ ± 0.033]	0.684 ± 0.075 [ ± 0.054]	0.899 ± 0.010 [ ± 0.007]	0.602 ± 0.030 [ ± 0.021]
Kaiko-ViT-S16	20×	0.892 ± 0.018 [ ± 0.013]	0.660 ± 0.043 [ ± 0.031]	0.764 ± 0.017 [ ± 0.012]	0.663 ± 0.040 [ ± 0.028]	0.676 ± 0.056 [ ± 0.040]	0.899 ± 0.011 [ ± 0.008]	0.603 ± 0.024 [ ± 0.017]
Kaiko-ViT-L14	5×	0.894 ± 0.017 [ ± 0.012]	0.646 ± 0.030 [ ± 0.021]	0.766 ± 0.020 [ ± 0.014]	0.656 ± 0.029 [ ± 0.021]	0.657 ± 0.045 [ ± 0.032]	0.899 ± 0.009 [ ± 0.007]	0.604 ± 0.026 [ ± 0.019]
Kaiko-ViT-B8	20×	0.894 ± 0.016 [ ± 0.011]	0.632 ± 0.043 [ ± 0.030]	0.756 ± 0.019 [ ± 0.014]	0.640 ± 0.042 [ ± 0.030]	0.656 ± 0.065 [ ± 0.047]	0.899 ± 0.010 [ ± 0.007]	0.590 ± 0.030 [ ± 0.022]
Kaiko-ViT-B16	20×	0.898 ± 0.016 [ ± 0.012]	0.657 ± 0.043 [ ± 0.030]	0.769 ± 0.019 [ ± 0.013]	0.666 ± 0.033 [ ± 0.024]	0.678 ± 0.063 [ ± 0.045]	0.902 ± 0.009 [ ± 0.007]	0.611 ± 0.028 [ ± 0.020]
H-Optimus-0	20×	0.899 ± 0.017 [ ± 0.012]	0.665 ± 0.046 [ ± 0.033]	0.775 ± 0.024 [ ± 0.017]	0.671 ± 0.042 [ ± 0.030]	0.675 ± 0.060 [ ± 0.043]	0.905 ± 0.011 [ ± 0.008]	0.621 ± 0.035 [ ± 0.025]
Hibou-L	5×	0.897 ± 0.018 [ ± 0.013]	0.664 ± 0.023 [ ± 0.017]	0.777 ± 0.018 [ ± 0.013]	0.668 ± 0.026 [ ± 0.019]	0.686 ± 0.034 [ ± 0.025]	0.908 ± 0.006 [ ± 0.004]	0.626 ± 0.023 [ ± 0.017]
Prov-Gigapath	20×	0.894 ± 0.016 [ ± 0.012]	0.675 ± 0.041 [ ± 0.029]	0.771 ± 0.024 [ ± 0.017]	0.680 ± 0.041 [ ± 0.029]	0.684 ± 0.056 [ ± 0.040]	0.903 ± 0.013 [ ± 0.009]	0.614 ± 0.035 [ ± 0.025]
CTransPath	10×	0.882 ± 0.013 [ ± 0.009]	0.647 ± 0.026 [ ± 0.018]	0.759 ± 0.020 [ ± 0.014]	0.644 ± 0.020 [ ± 0.015]	0.670 ± 0.042 [ ± 0.030]	0.902 ± 0.008 [ ± 0.005]	0.597 ± 0.028 [ ± 0.020]
CONCHv1.5	20×	0.889 ± 0.015 [ ± 0.011]	0.646 ± 0.031 [ ± 0.022]	0.748 ± 0.018 [ ± 0.013]	0.657 ± 0.024 [ ± 0.017]	0.668 ± 0.052 [ ± 0.037]	0.895 ± 0.007 [ ± 0.005]	0.578 ± 0.024 [ ± 0.017]
CONCH	10×	0.891 ± 0.017 [ ± 0.012]	0.676 ± 0.021 [ ± 0.015]	0.770 ± 0.015 [ ± 0.011]	0.684 ± 0.014 [ ± 0.010]	0.695 ± 0.048 [ ± 0.035]	0.901 ± 0.008 [ ± 0.006]	0.612 ± 0.018 [ ± 0.013]
Ensemble	Multi	**0.907** ± **0.017 [** ± **0.012]**	**0.696** ± **0.033 [** ± **0.024]**	**0.795** ± **0.017 [** ± **0.012]**	**0.708** ± **0.025 [** ± **0.018]**	**0.697** ± **0.050 [** ± **0.035]**	**0.911** ± **0.008 [** ± **0.005]**	**0.651** ± **0.024 [** ± **0.018]**

*SD* standard deviation *CI* confidence interval *HW* half-width.

Bold values indicate the top-performing result for each metric.

Class-wise analysis revealed asymmetric misclassification in dysplastic-related classes. (Figure S1) While the non-Barrett/normal and non-dysplastic Barrett’s lesion categories showed higher recall, the dysplastic tissue category was undercalled by models. Models produced a higher false negative rate for dysplastic and cancerous categories than for normal or metaplastic categories. Misclassification predominantly occurred between morphologically adjacent categories, particularly among non-dysplastic Barrett’s, dysplastic Barrett’s, and EAC. Greater classification errors clustered around the same lesion categories that are known to exhibit the highest interobserver variability among pathologists^26^. However, the results showed that most models performed better at 20× magnification (Table 1), suggesting that models at this magnification may provide discriminative morphological information for morphological subtyping.

To provide an overview of concordance among the models in morphological subtyping, we evaluated pairwise agreement. Results indicated that integrating Virchow2, UNI2-h, Hibou-L, Virchow, or the UNI backbone with the N2EAC-MIL model yielded the highest level of concordance with the expert consensus diagnosis used as the reference standard for BE morphological subtyping (κ ± SD: 0.638 ± 0.017 [95% CI ± 0.012], 0.628 ± 0.023 [95% CI ± 0.016], 0.626 ± 0.023 [95% CI ± 0.017], 0.624 ± 0.034 [95% CI ± 0.024], 0.622 ± 0.028 [95% CI ± 0.020]) (Fig. 1c; Table 1). Among these five expert-consistent (ExCo) models, the Virchow2 model achieved the highest agreement with the reference standard, while CONCHv1.5 achieved the lowest agreement (κ ± SD: 0.578 ± 0.024 [95% CI ± 0.017]). ExCo models were defined as those that achieved the highest concordance with the reference standard among all single-scale models. The results showed higher and more consistent concordance between FMs than between FMs and the reference standard (Fig. 1c; Table 1). Moreover, multi-rater agreement evaluation yielded a mean Fleiss’ κ of 0.779 ± 0.023 across all raters, including the reference standard and the models.

### Cancer detection

**Fig. 2:**
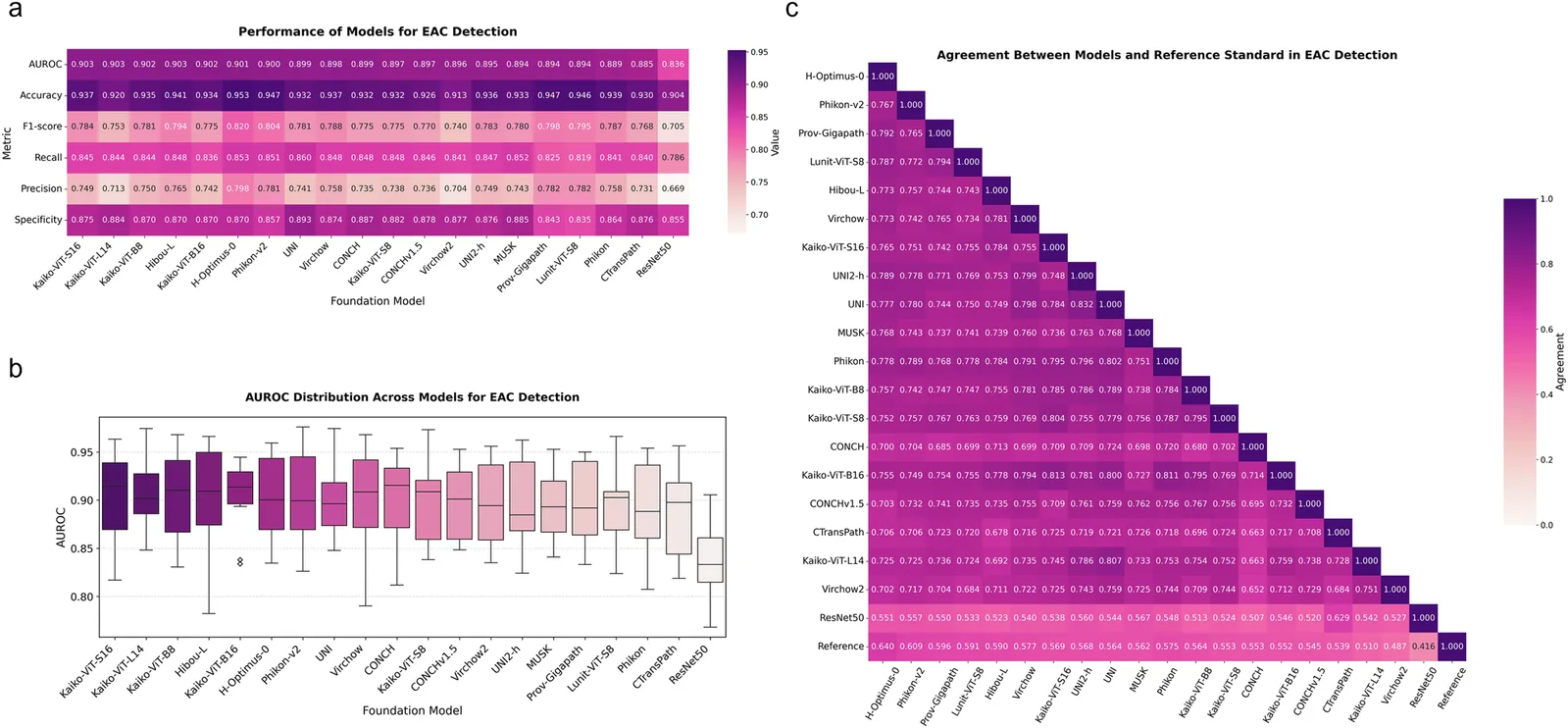
Models’ performance evaluation for EAC detection. **a** Heatmap illustrating N2EAC-MIL models’ performance for EAC detection; **b** Each box plot summarizes the distribution of mean AUROC across all folds. The boxes are sorted based on the mean scores of the models; **c** Pairwise agreement between N2EAC-MIL models and the reference standard.

When we integrated the N2EAC-MIL model with Kaiko-ViT-S16 or Kaiko-ViT-L14, we obtained a strong overall discriminative performance (Fig. 2a, AUROC: 0.903 ± 0.046 [95% CI ± 0.033], AUROC: 0.903 ± 0.038 [95% CI ± 0.027], respectively). It is worth noting that H-Optimus-0 achieved the highest F1-score (0.820 ± 0.037 [95% CI ± 0.026]) among all evaluated models (Fig. 2a; Table 2). In the EAC detection task, high macro recall was achieved by N2EAC-MIL models when integrated with Histo-FMs (recall ≥ 0.819) (Table 2). While the CONCH model showed a higher median AUROC in the distribution plots, its overall mean AUROC was not among the top-performing models, suggesting variability across the cross-validation folds (Fig. 2b). Table S2 and Fig. S3–S4 provide more details about the models’ performance profile.

**Table 2:** Performance metrics for EAC detection

Foundation Model	Magnification	AUROC Mean ± SD [95% CI HW]	F1-Score Mean ± SD [95% CI HW ]	Accuracy Mean ± SD [95% CI HW]	Precision Mean ± SD [95% CI HW]	Recall Mean ± SD [95% CI HW]	Specificity Mean ± SD [95% CI HW]	Cohen’s Kappa κ ± SD [95% CI HW]
Virchow2	40×	0.896 ± 0.046 [ ± 0.033]	0.740 ± 0.041 [ ± 0.029]	0.913 ± 0.021 [ ± 0.015]	0.704 ± 0.057 [ ± 0.041]	0.841 ± 0.051 [ ± 0.036]	0.877 ± 0.073 [ ± 0.052]	0.487 ± 0.079 [ ± 0.056]
Virchow	40×	0.898 ± 0.054 [ ± 0.038]	0.788 ± 0.046 [ ± 0.033]	0.937 ± 0.023 [ ± 0.016]	0.758 ± 0.064 [ ± 0.045]	0.848 ± 0.049 [ ± 0.035]	0.874 ± 0.051 [ ± 0.037]	0.577 ± 0.091 [ ± 0.065]
UNI2-h	40×	0.895 ± 0.049 [ ± 0.035]	0.783 ± 0.041 [ ± 0.029]	0.936 ± 0.017 [ ± 0.012]	0.749 ± 0.046 [ ± 0.033]	0.847 ± 0.054 [ ± 0.038]	0.876 ± 0.057 [ ± 0.041]	0.568 ± 0.081 [ ± 0.058]
UNI	40×	0.899 ± 0.040 [ ± 0.029]	0.781 ± 0.048 [ ± 0.034]	0.932 ± 0.021 [ ± 0.015]	0.741 ± 0.054 [ ± 0.039]	**0.860** ± **0.041 [** ± **0.029]**	**0.893** ± **0.038 [** ± **0.027]**	0.564 ± 0.093 [ ± 0.067]
ResNet50	10×	0.836 ± 0.045 [ ± 0.032]	0.705 ± 0.034 [ ± 0.024]	0.904 ± 0.019 [ ± 0.013]	0.669 ± 0.028 [ ± 0.020]	0.786 ± 0.052 [ ± 0.037]	0.855 ± 0.040 [ ± 0.029]	0.416 ± 0.065 [ ± 0.047]
Phikon-v2	20×	0.900 ± 0.051 [ ± 0.036]	0.804 ± 0.041 [ ± 0.029]	0.947 ± 0.011 [ ± 0.008]	0.781 ± 0.051 [ ± 0.037]	0.851 ± 0.069 [ ± 0.049]	0.857 ± 0.099 [ ± 0.071]	0.609 ± 0.082 [ ± 0.058]
Phikon	40×	0.889 ± 0.051 [ ± 0.037]	0.787 ± 0.042 [ ± 0.030]	0.939 ± 0.017 [ ± 0.012]	0.758 ± 0.050 [ ± 0.036]	0.841 ± 0.056 [ ± 0.040]	0.864 ± 0.064 [ ± 0.046]	0.575 ± 0.082 [ ± 0.059]
MUSK	20×	0.894 ± 0.040 [ ± 0.029]	0.780 ± 0.051 [ ± 0.037]	0.933 ± 0.021 [ ± 0.015]	0.743 ± 0.059 [ ± 0.042]	0.852 ± 0.048 [ ± 0.034]	0.885 ± 0.045 [ ± 0.032]	0.562 ± 0.101 [ ± 0.072]
Lunit-ViT-S8	10×	0.894 ± 0.041 [ ± 0.030]	0.795 ± 0.068 [ ± 0.049]	0.946 ± 0.020 [ ± 0.014]	0.782 ± 0.078 [ ± 0.056]	0.819 ± 0.064 [ ± 0.046]	0.835 ± 0.074 [ ± 0.053]	0.591 ± 0.135 [ ± 0.097]
Kaiko-ViT-S8	20×	0.897 ± 0.045 [ ± 0.032]	0.775 ± 0.045 [ ± 0.032]	0.932 ± 0.018 [ ± 0.013]	0.738 ± 0.051 [ ± 0.037]	0.848 ± 0.051 [ ± 0.037]	0.882 ± 0.055 [ ± 0.039]	0.553 ± 0.089 [ ± 0.063]
Kaiko-ViT-S16	40×	0.903 ± 0.046 [ ± 0.033]	0.784 ± 0.038 [ ± 0.027]	0.937 ± 0.017 [ ± 0.012]	0.749 ± 0.045 [ ± 0.032]	0.845 ± 0.049 [ ± 0.035]	0.875 ± 0.056 [ ± 0.040]	0.569 ± 0.075 [ ± 0.053]
Kaiko-ViT-L14	20×	0.903 ± 0.038 [ ± 0.027]	0.753 ± 0.033 [ ± 0.024]	0.920 ± 0.021 [ ± 0.015]	0.713 ± 0.037 [ ± 0.026]	0.844 ± 0.041 [ ± 0.029]	0.884 ± 0.038 [ ± 0.027]	0.510 ± 0.062 [ ± 0.045]
Kaiko-ViT-B8	20×	0.902 ± 0.048 [ ± 0.034]	0.781 ± 0.045 [ ± 0.033]	0.935 ± 0.020 [ ± 0.015]	0.750 ± 0.061 [ ± 0.044]	0.844 ± 0.049 [ ± 0.035]	0.870 ± 0.059 [ ± 0.042]	0.564 ± 0.089 [ ± 0.064]
Kaiko-ViT-B16	40×	0.902 ± 0.038 [ ± 0.028]	0.775 ± 0.038 [ ± 0.027]	0.934 ± 0.019 [ ± 0.013]	0.742 ± 0.046 [ ± 0.033]	0.836 ± 0.042 [ ± 0.030]	0.870 ± 0.045 [ ± 0.032]	0.552 ± 0.075 [ ± 0.053]
H-Optimus-0	40×	0.901 ± 0.045 [ ± 0.032]	0.820 ± 0.037 [ ± 0.026]	0.953 ± 0.010 [ ± 0.007]	0.798 ± 0.049 [ ± 0.035]	0.853 ± 0.038 [ ± 0.027]	0.870 ± 0.051 [ ± 0.037]	0.640 ± 0.073 [ ± 0.052]
Hibou-L	40×	0.903 ± 0.058 [ ± 0.042]	0.794 ± 0.041 [ ± 0.029]	0.941 ± 0.019 [ ± 0.014]	0.765 ± 0.051 [ ± 0.037]	0.848 ± 0.053 [ ± 0.038]	0.870 ± 0.072 [ ± 0.052]	0.590 ± 0.081 [ ± 0.058]
Prov-Gigapath	10×	0.894 ± 0.046 [ ± 0.033]	0.798 ± 0.052 [ ± 0.037]	0.947 ± 0.016 [ ± 0.011]	0.782 ± 0.065 [ ± 0.046]	0.825 ± 0.050 [ ± 0.036]	0.843 ± 0.054 [ ± 0.039]	0.596 ± 0.103 [ ± 0.074]
CTransPath	5×	0.885 ± 0.045 [ ± 0.032]	0.768 ± 0.039 [ ± 0.028]	0.930 ± 0.017 [ ± 0.012]	0.731 ± 0.041 [ ± 0.029]	0.840 ± 0.051 [ ± 0.037]	0.876 ± 0.055 [ ± 0.039]	0.539 ± 0.076 [ ± 0.054]
CONCHv1.5	20×	0.897 ± 0.041 [ ± 0.029]	0.770 ± 0.055 [ ± 0.040]	0.926 ± 0.033 [ ± 0.023]	0.736 ± 0.061 [ ± 0.043]	0.846 ± 0.046 [ ± 0.033]	0.878 ± 0.046 [ ± 0.033]	0.545 ± 0.105 [ ± 0.075]
CONCH	40×	0.899 ± 0.047 [ ± 0.034]	0.775 ± 0.039 [ ± 0.028]	0.932 ± 0.016 [ ± 0.011]	0.735 ± 0.042 [ ± 0.030]	0.848 ± 0.040 [ ± 0.029]	0.887 ± 0.034 [ ± 0.024]	0.553 ± 0.076 [ ± 0.054]
Ensemble	Multi	**0.909** ± **0.052 [** ± **0.037]**	**0.836** ± **0.043 [** ± **0.031]**	**0.959** ± **0.012 [** ± **0.009]**	**0.832** ± **0.063 [** ± **0.045]**	0.853 ± 0.055 [ ± 0.039]	0.848 ± 0.089 [ ± 0.064]	**0.673** ± **0.087 [** ± **0.062**]

*SD* standard deviation *CI* confidence interval *HW* half-width.

Bold values indicate the top-performing result for each metric.

Interestingly, in this task, we observed that the Prov-Gigapath model, with the DINOv2 framework^42^ achieved the highest inter-model concordance with H-Optimus-0 which is a pretrained Histo-FM based on the iBOT framework^43^ and DINOv2. Similar downstream classification behavior was observed despite the use of different pretraining configuration.

The multi-rater agreement assessment yielded a Fleiss’ κ of 0.722 ± 0.037, indicating substantial agreement among all models and the reference standard. Five ExCo models in EAC detection included H-Optimus-0 (κ ± SD: 0.640 ± 0.073 [95% CI ± 0.052]), Phikon-v2 (0.609 ± 0.082 [95% CI ± 0.058]), Prov-Gigapath (0.596 ± 0.103 [95% CI ± 0.074]), Lunit-ViT-S8 (0.591 ± 0.135 [95% CI ± 0.097]), and Hibou-L (0.590 ± 0.081 [95% CI ± 0.058]) (Fig. 2c).

### Cross-task performance comparison

The N2EAC-MIL model achieved a median AUROC of 0.88 to 0.91 for both tasks across all backbones (Fig. S5). The model demonstrated stronger performance on EAC detection than on morphological subtyping, suggesting that N2EAC-MIL exhibited more consistent EAC/non-EAC discrimination. Although the models were more consistent in cancer detection, yielding lower performance variability, morphological subtyping in a heterogeneous, imbalanced dataset remained more challenging.

### Impact of magnification level on diagnostic model performance

**Fig. 3:**
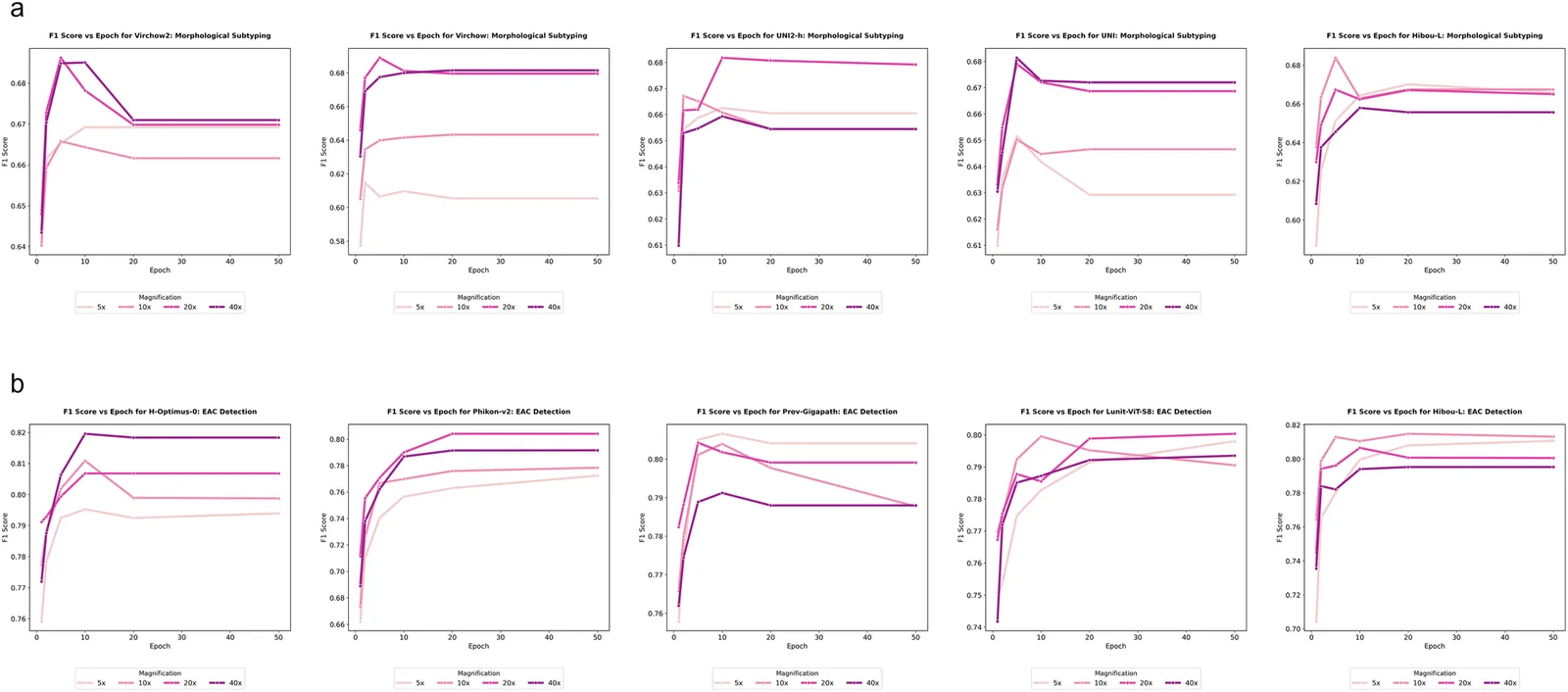
F1 score trends of ExCo models across magnification levels and max epochs. **a** From left to right, F1 score trend across models integrated with Virchow2, Virchow, UNI, UNI2-h, and Hibou-L for the subtyping task. Compared to other models, the N2EAC-MIL model, when integrated with Hibou-L, achieves a higher F1 score at 10× magnification than at higher magnifications. In the case of standardizing magnification, Virchow or Uni2-h can achieve a higher, more balanced precision-recall score for morphological subtyping. **b** From left to right, F1 score across models paired with H-Optimus-0, Phikon-v2, Prov-Gigapath, Lunit-ViT-S8, and Hibou-L for EAC detection. Among all cancer detection models, H-Optimus-0 achieves the highest F1 score (0.820* ±* 0.037 [95% CI* ±* 0.026]) at 40× magnification.

Figure 3a illustrates the trend in the ExCo models’ F1 scores across diverse magnifications and training durations in the morphological subtyping task. Most subtyping models reached a plateau within approximately 10 epochs, indicating that models generally converged early (within 5 to 10 epochs). In most morphological subtyping experiments, the model achieved higher performance when features were extracted at 20× and 40× magnification levels (Table 1). The F1 score trends for other models are shown in Fig. S6.

Figure 3b shows the F1 scores across ExCo models in the cancer detection task. Results showed that most models achieved a high overall AUROC at 40×. Different Histo-FMs behaved differently in terms of F1-score at 40× magnification (Fig. S7). H-Optimus-0 achieved the highest F1 score at the 40× magnification level. Notably, Hibou-L demonstrated high performance across both diagnostic tasks.

### Pathologist validation

**Fig. 4:**
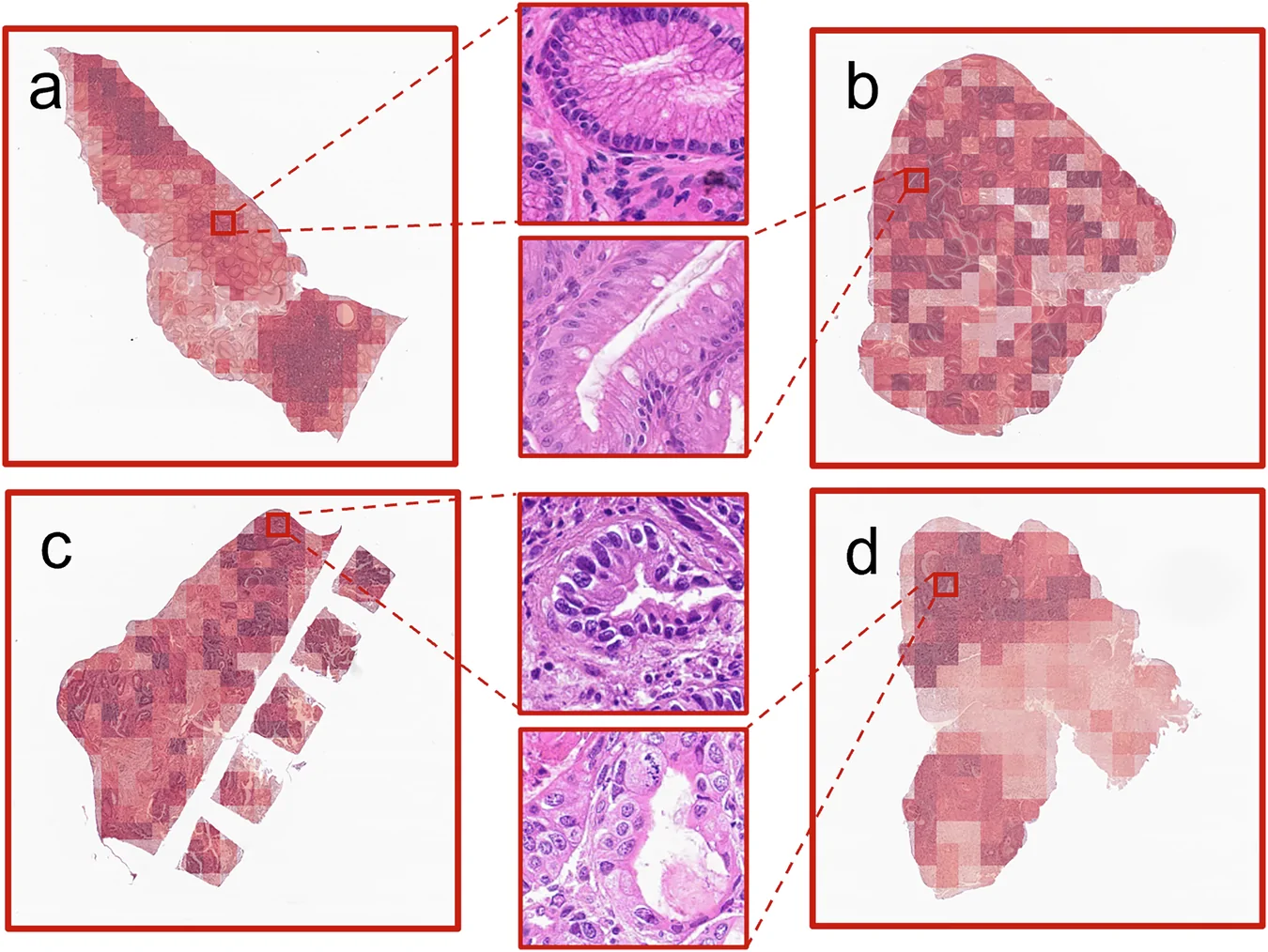
Representative diagnostically relevant patch from each subtype. **a** Cardiac mucosa showing normal gastric-type epithelium; **b** Non-dysplastic Barrett’s mucosa containing intestinal-type columnar epithelium with goblet cells; **c** Dysplastic Barrett’s mucosa with a high-attention patch showing dysplastic glands; **d** Adenocarcinoma and squamous epithelium with a high-attention patch illustrating invasive tumor glands.

To compare the spatial localization of ExCo models’ output, attention heatmaps of the same four slides across all models, one from non-Barrett/normal tissue, Barrett’s metaplasia without dysplasia, Barrett’s metaplasia with dysplasia, and adenocarcinoma were generated. Figure S8 shows all attention maps across five top ExCo models were reviewed by a board-certified pathologist who participated in the original diagnostic assessment of the cohort, with the heatmaps focusing exclusively on the spatial correspondence between highlighted regions and diagnostically informative morphology. The assessment results showed heterogeneous localization profiles across models, with some producing more focused localization and others producing broader attention distributions. According to our observations, although partial coverage of diagnostically relevant regions was highlighted in the evaluated slides, the models did not fully capture all relevant structures. Figure 4 shows four examples of model output at 20×, one for each class, demonstrating slides classified by the ExCo models in the morphological subtyping task.

In a separate multi-rater heatmap assessment, 35 heatmaps, derived from predictions of 5 ExCo models, were generated at magnification levels selected based on quantitative performance on the morphological subtyping task (Table 1; Fig. S9). For each heatmap, the pathologists assigned a score to the alignment between the heatmap and diagnostically relevant tissue regions, with a mean ± SD of 3.467 ± 0.961 (out of 5) and a median of 3.667 (IQR: 2.833–4.333). Qualitative inspection indicated that the models’ attention tended to be localized to diagnostically informative morphological structures, although it was also partially distributed across non-target tissue regions. However, some limitations were observed, including reduced attention to dysplastic morphology and an overemphasis on non-diagnostic structures, such as necrosis, inflammation, fibrin, and stroma. Inter-rater reliability assessment showed moderate agreement (ICC(A,k) = 0.651, 95% CI: 0.37–0.81) among reviewers on the alignment of heatmaps with diagnostically informative regions. Despite occasional attention failures and localization errors, the results indicated that the models’ attention was partially localized to diagnostically relevant morphology in dysplastic and adenocarcinoma lesions.

### Ensemble

**Fig. 5:**
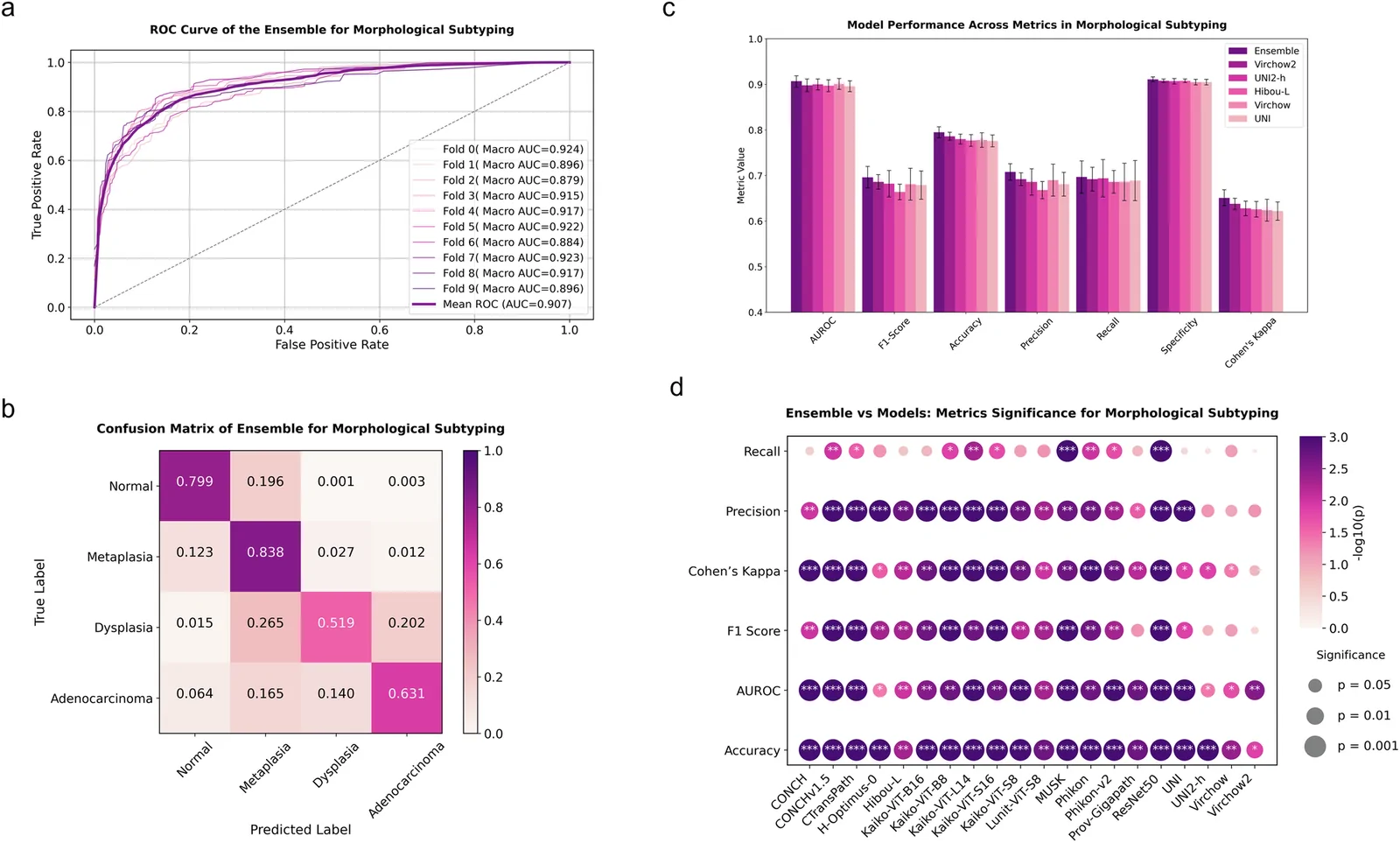
Performance of multi-backbone and multi-magnification ensemble for morphological subtyping. **a** ROC curve of ensemble; **b** average confusion matrix across folds; **c** Top Five ExCo Models and Ensemble Performance, ranked by Cohen’s Kappa; **d** Significance of ensemble vs. individual models across metrics. Statistical significance level (−log10(p)) obtained from a one-sided paired Wilcoxon signed-rank test across 10 Monte Carlo cross-validation runs. Circle size and its color represent the statistical significance level, and asterisks denote a significant improvement of the ensemble over the model (*p < 0.05, **p < 0.01, ***P < 0.001).

Compared with single-scale models, aggregating the ExCo models yielded a higher F1 score of 0.696 ± 0.033 [95% CI ± 0.024], an AUROC of 0.907 ± 0.017 [95% CI ± 0.012], and an accuracy of 0.795 ± 0.017 [95% CI ± 0.012] for morphological subtyping (Fig. 5a; Table 1). The N2EAC approach for the subtyping task yielded per-class recall of 0.799 ± 0.033 [95% CI ± 0.023] for the Non-Barrett’s normal tissue class, 0.838 ± 0.029 [95% CI ± 0.021] for the Barrett’s metaplasia without dysplasia class, 0.519 ± 0.176 [95% CI ± 0.126] for the Barrett’s metaplasia with dysplasia class, and 0.631 ± 0.093 [95% CI ± 0.066] for the adenocarcinoma class (Fig. 5b). Findings showed that although aggregating the ExCo models improved discrimination relative to many single-scale models (Fig. 5c), it did not significantly improve recall across all models (Fig. 5d).

Although ensembling ExCo models with diverse magnification levels and backbones did not show a statistically significant improvement in agreement with the reference standard compared with the top ExCo model (Virchow2)(*p* = 0.138), the finding showed an improvement in agreement with the reference standard across all other single-scale models (*p* < 0.05) in morphological subtyping. Findings demonstrated that ensembling the top five ExCo-N2EAC-MIL models significantly outperformed most single-scale models in terms of AUROC and accuracy (Fig. 5d). While ensembling improved prediction performance, its advantages were not uniform across metrics.

**Fig. 6:**
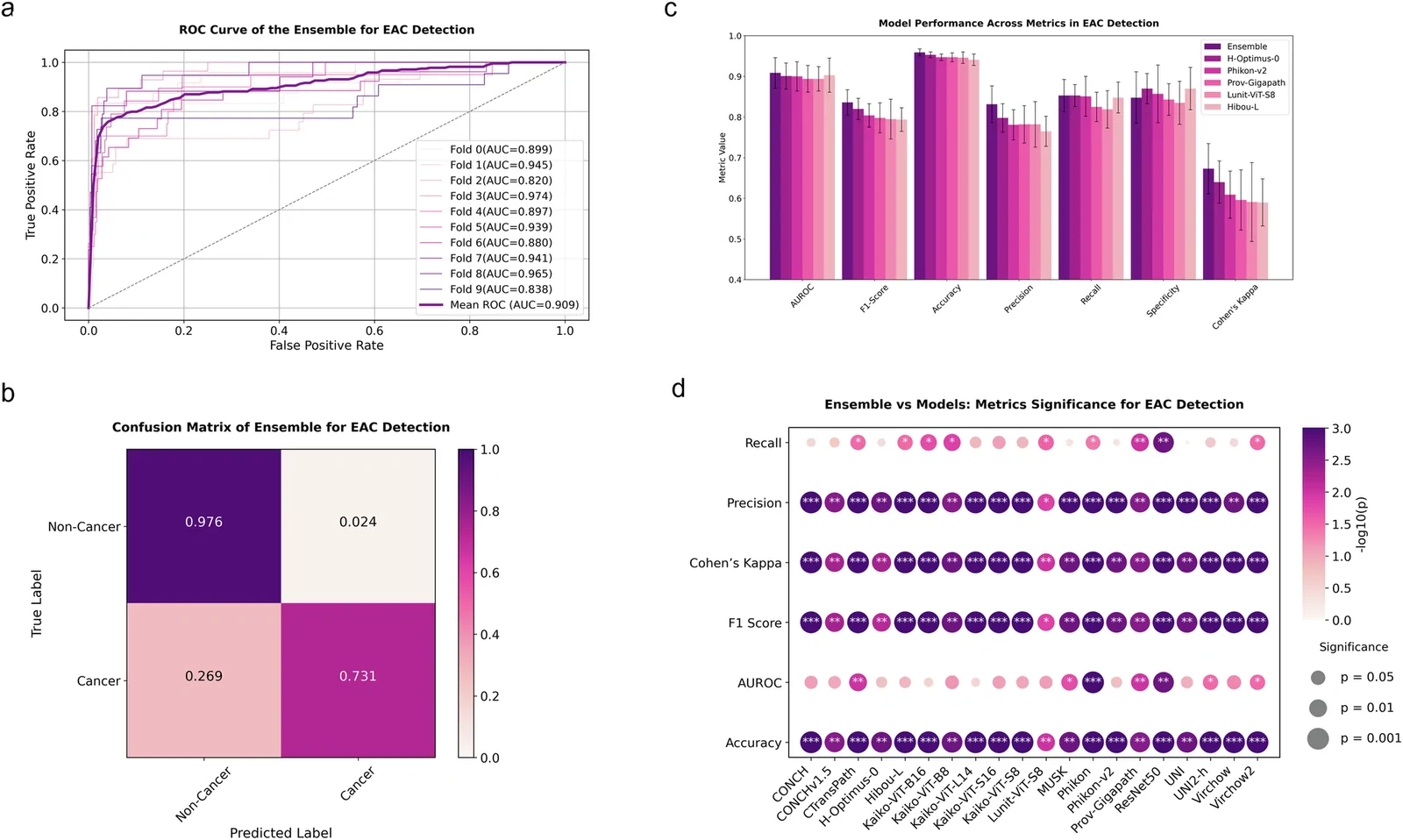
Performance of multi-backbone and multi-magnification ensemble for EAC detection. **a** ROC curve of ensemble; **b** average confusion matrix across folds; **c** The highest AUROC scores in the EAC detection task were achieved by models utilizing Kaiko-ViT-B16, Hibou-L, Kaiko-ViT-S16, Kaiko-ViT-L14, and Kaiko-ViT-B8, outperforming other models; **d** Significance of ensemble vs. single models across metrics. Statistical significance level (−log10(p)) obtained from a one-sided paired Wilcoxon signed-rank test across the 10 Monte Carlo cross-validation runs. Circle size and its color represent the statistical significance level, and asterisks denote a significant improvement of the ensemble over the model (*p < 0.05, **p < 0.01, ***p < 0.001).

For cancer detection, compared with single-scale models, the multi-magnification ensemble achieved a higher F1 score of 0.836 ± 0.043 [95% CI ± 0.031], AUROC of 0.909 ± 0.052 [95% CI ± 0.037], and accuracy of 0.959 ± 0.012 [95% CI ± 0.009] for cancer detection (Fig. 6a; Table 2). We achieved per-class recall of 0.976 ± 0.013 [95% CI ± 0.009] for the non-cancer class and 0.731 ± 0.114 [95% CI ± 0.081] for the cancer class (Fig. 6b). An ensemble of the top five ExCo models demonstrated significantly higher agreement (0.673 ± 0.087 [95% CI ± 0.062], *p* < 0.01) with the reference standard than any individual model, illustrating the ensemble’s ability to achieve higher classification concordance (Fig. 6c, d). The ensemble of the top five ExCo models showed a statistically significant improvement over Lunit-ViT-S8 (*p* < 0.05) and Prov-Gigapath (*p* < 0.01), and Hibou-L (*p* < 0.05) in terms of recall (Fig. 6c, d).

The results indicated that the ensemble model outperformed the EAC single-scale models in most evaluation criteria, including accuracy, Cohen’s Kappa, F1 score, and precision (with *p* < 0.01 in most model comparisons) (Fig. 6d), though this improvement was not uniform across models. The agreement with the reference standard improved from 0.640 ± 0.073 [95% CI ± 0.052] in the top ExCo model to 0.673 ± 0.087 [95% CI ± 0.062]. However, the improvement in AUROC after ensembling models was not statistically significant compared to the performance of most individual models (*p* > 0.05), indicating no strong, uniform superiority in overall discrimination between malignant and non-malignant lesions. In total, the findings showed that ensemble benefit was metric- and model-dependent, as well as task-dependent.

## Discussion

Barrett’s esophagus is a major precursor lesion of esophageal adenocarcinoma; however, only a minority of cases progress to cancer, while diagnostic assessment is complicated by substantial interobserver variability. The pathological BE continuum is characterized by considerable heterogeneity in morphology, tissue quality, and diagnostic interpretation. The known interobserver variability^26^ and morphological overlap in BE dysplasia grading and borderline lesions limit diagnostic reproducibility. This challenge is particularly evident in the distinction between non-dysplastic Barrett’s mucosa, dysplasia, and early neoplasia, where morphological overlap is present along the progression continuum. To enable reproducibility and method development, controlled benchmark datasets are generally used; however, they may fail to capture the complexity of clinical practice and the inherent variability of disease. In contrast, the use of an inherently heterogeneous and imbalanced cohort better reflects the routine diagnostic spectrum encountered in practice.

Histo-FMs are designed with the primary objective of learning transferable morphological representations that are intended to facilitate downstream transfer and adaptability to pre-analytical variation, and enable cross-domain portability^3,8,44^. Benchmarking emerging foundation models for specific tasks outside well-curated public datasets is limited^8^. This study provides a systematic benchmark for evaluating the application of Histo-FMs to specific tasks across a progression continuum. The findings highlighted promising morphological discrimination across progression-related categories in a heterogeneous cohort. Despite relative confusion in distinguishing non-dysplastic from dysplastic tissue, some models retained greater sensitivity to dysplastic morphologic features, motivating further evaluation of Histo-FMs for the recognition of diagnostically ambiguous lesions and supporting further investigation of AI-based pathology-aid workflows. From a pathological perspective, such approaches may be particularly valuable for decision support and for augmenting pathological assessment of diagnostically challenging BE lesions, rather than replacing expert pathological interpretation.

The variable performance of single-scale models in predicting dysplasia suggests that different Histo-FMs may capture complementary predictive patterns. While a single model offers simpler deployment, lower computational cost, and easier maintenance, task-tailored ensemble strategies, such as aggregating expert-consistent morphological subtyping models, may leverage complementary predictive behavior and yield incremental performance gains. Therefore, based on the findings in this study, model selection should remain task-dependent. When operational complexity should be minimized, and incremental benefits from ensembling are limited, a single model may be prioritized. However, when the target task benefits from complementary model behavior and the ensemble mitigates model-specific errors, the task-specific ensemble may be considered selectively. The study showed recall gains were comparatively limited, and performance gains varied across classes. Therefore, for the dysplasia-associated lesions, the ensemble showed a residual recall limitation despite aggregation. For a dysplasia-focused workflow, model selection should account for residual class-specific failure modes.

Uncertainty in the grading of BE-associated dysplasia remains a longstanding diagnostic challenge due to the biological and morphological continuum of neoplastic progression. Subtle morphology, interobserver variability, and class imbalance are likely contributors to lower recall rates in dysplastic and cancerous samples. High interobserver variability in grading LGD and HGD, even among gastrointestinal pathology experts, has been reported^26,45,46^. Consistent with this clinical reality, the lower recall observed in dysplastic lesions may reflect the inherent morphological ambiguity and the known interobserver variability associated with this diagnostic category. In line with the known interobserver variability in practice, one of the most clinically meaningful findings is that models’ confusion was greater in categories associated with higher interobserver variability^26^. Misclassification was observed primarily between non-dysplastic Barrett’s mucosa, dysplasia, and adenocarcinoma, whereas higher recall was observed for predicting adenocarcinoma. The pattern shows that model misclassification is concentrated in morphologically transitional lesion categories, underscoring the inherent diagnostic complexity of BE-associated dysplasia. This pattern was consistent across most models and persisted after ensembling, suggesting that the principal challenge centered around morphologically transitional stages. The observed prediction errors in morphological subtyping may reflect overlapping histomorphological features and the inherent diagnostic ambiguity of BE-associated dysplasia.

The qualitative pathologist-guided evaluation indicated morphological plausibility, with moderate alignment with diagnostically informative regions and pathology-consistent attention patterns. The pathologists’ review shows that ExCo models tended to localize to diagnostically relevant dysplastic and neoplastic morphology along the Barrett’s progression continuum. Although models highlighted dysplasia-relevant morphology despite morphological heterogeneity, in some cases these regions were incompletely localized, and attention was distributed to non-diagnostic structures, such as inflammatory, stromal, necrotic, and fibrinous structures. Notably, even in some misclassified cases, attention maps highlighted histomorphologically relevant regions, suggesting that models’ confusion may arise not only from failure to identify diagnostically informative tissue but also from difficulty categorizing lesions. The qualitative spatial localization assessment indicates moderate inter-rater agreement in heatmap morphology alignment and is consistent with histomorphologically relevant localization behavior in models. The findings suggest that attention heatmaps may provide spatial localization information for diagnostically difficult lesions.

Furthermore, PFPE was introduced as an alternative to address the molecular limitations of conventional FFPE processing and to preserve molecular integrity compared with conventional formalin fixation^26,27^. Previous comparative studies have demonstrated diagnostic comparability between PFPE and FFPE for routine histopathological assessment^26^. To the best of our knowledge, current Histo-FMs are pretrained on WSIs with fixation types other than PFPE. In addition, given that PFPE is not the routine clinical fixation standard, PFPE datasets are comparatively scarce^47^ and may constrain the development of PFPE-specific FMs. Fixation-related variation in tissue appearance, shift in visual distribution, and fixation-induced domain shift may constitute a distinct evaluation setting. Although the aim of this study was not to develop a PFPE-specific image-processing pipeline, it contributed to a systematic assessment of Histo-FMs’ behavior in a PFPE setting. A matched PFPE-FFPE comparison was not available in the present cohort; cross-fixation transferability remains to be validated in future studies. Nevertheless, the observed performance provides initial evidence for the feasibility of Histo-FM use in the PFPE setting and supports further investigation into cross-fixation transferability. Furthermore, the observed potential of Histo-FM application motivates further investigation into multimodal and integrated morphology-molecular computational pathology workflows.

The findings suggest inter-model concordance in predictions among Histo-FMs. The substantial concordance with the reference standard across ExCo models suggests that diagnostically relevant predictive patterns may be captured in the morphological subtyping task. The observed pairwise agreement between the Histo-FMs suggests consistent downstream classification behavior across models in this study, especially within the same family (e.g., UNI and UNI2-h). In contrast, lower agreement between the models and the reference standard may partly reflect class imbalance, model limitations, and morphological overlap across BE progression stages. Although ExCo models are imperfectly aligned with the reference standard, they may produce similar downstream decision patterns. Further studies should be conducted to investigate the representation-level basis underlying the observed similarities in predictions between Histo-FMs.

Increasing diagnostic workload and pathologist workforce constraints^48,49^, the scarcity of subspecialty expertise in assessing BE biopsies^49,50^ and the known interobserver variability^51^ in assessing BE-associated lesions may plausibly contribute to prolonged review times and may create challenges in prioritizing high-risk lesions^16,52^ in the Barrett progression spectrum, and may add pressure on diagnostic workflows^50^. A plausible clinical role for the Histo-FMs-based ensemble approach may be to support pathologists in BE lesion assessments within an assistive framework, which warrants dedicated evaluation of workflow efficiency and also requires prospective assessment of expert-review allocation. The study is a research-stage evaluation of Histo-FM ensembling for Barrett’s esophagus lesions, while clinical integration of an AI-driven ensemble approach that supports pathologists in diagnosis was beyond the scope of the present study and remains to be explored. Therefore, from a translational perspective, by providing complementary diagnostic information and serving as an adjunctive tool, the Histo-FM ensemble approach may prompt secondary review. However, our study showed heterogeneous gains from ensembling. Additionally, inter-model prediction discordance as a candidate triage signal could be explored as a future prioritization mechanism, which may help direct expert attention to diagnostically challenging cases and assist in flagging them for additional expert review.

The complexity of subjective diagnostic assessment, particularly when encountering transitional or borderline lesions, may lead to inconsistent grading and diagnosis of dysplasia. According to the current study’s findings, classifying lesions along the dysplasia-neoplasia spectrum remained challenging, with persistent difficulty in dysplasia classification. Remaining limitations in dysplasia recall support the rationale for an expert-in-the-loop strategy in response to the challenges posed by morphologically transitional lesions. The findings suggest that aggregating models’ probabilities can increase their ability to detect non-dysplastic cases, while a subset of dysplastic lesions remains undetected. Despite failing to detect a proportion of dysplastic lesions, selected global and class-specific performance metrics were improved after ensembling ExCo models. This residual limitation defines an important boundary for the complementary clinical role of the Histo-FM-driven ensemble approach: while AI could provide complementary second-reader support, ambiguous and dysplastic cases should retain expert oversight. In practical terms, using the Histo-FM-based ensembling approach to flag diagnostically challenging lesions could support future prioritization of cases within transitional categories that warrant closer pathological review and expert assessment. The Histo-FM-assisted approach may be better positioned within a human-AI collaboration workflow than as an autonomous diagnostic tool.

Despite the observed limitation in dysplasia recall along the BE progression continuum, spatial localization of potentially relevant regions could help pathologists inspect model-attended regions and provide complementary spatial information. The visualization can also reveal localization failure modes, potentially helping assess whether model-attended regions are morphologically plausible and supporting critical appraisal of model outputs. The heatmaps, if implemented in the workflow, may help highlight candidate regions for closer inspection of dysplastic features by pathologists. Therefore, in a workflow-support scenario, the approach may facilitate expert reassessment of borderline dysplastic lesions rather than serve as a standalone decision-maker within the pathology setting.

However, the present study should be interpreted as a retrospective benchmarking study rather than as evidence of immediate clinical adoption. Nonetheless, broader clinical translation requires external validation of the approach in independent, routine FFPE multi-site cohorts to assess the generalizability to routine FFPE and multi-scanner settings. Routine FFPE generalizability has not been demonstrated in the current study; cross-fixation transferability from PFPE to FFPE remains uncertain. Moreover, due to the limited representation of dysplasia cases in this study, dysplasia-enriched FFPE validation should be prioritized. In addition, future studies should investigate the approach’s effectiveness in expert-review allocation and triage performance to determine its value within the dysplasia review workflow.

The study has limitations: it includes only a specific cohort and tumor type, and some aspects of Histo-FM evaluation could not be addressed in the current work. All slides were digitized using a single scanner type and a single acquisition pipeline, as generalization across scanners was not part of this proof-of-concept study. Future inclusion of scanner diversity will enable investigation of scanner-induced domain shift in BE morphological subtyping and EAC classification. Moreover, although we included a broad range of Histo-FMs in the current study, we were limited to models available under research-use licenses. Therefore, evaluating the applicability of the other models in BE data remains to be explored. Furthermore, we assessed the applicability of the Histo-FMs in PFPE tissue in a fixation-specific cohort; cross-fixation generalization was not evaluated, and the applicability of our approach to conventional FFPE workflows remains to be externally validated on large-scale, multi-institutional, multi-scanner routine FFPE cohorts. Therefore, future studies should validate the robustness and transferability across standard FFPE workflows and clinical settings before routine clinical deployment. In addition, most Histo-FMs are pre-trained at specific magnification levels (20× is the most commonly used), and may not capture multi-magnification contextual cues. Although we aggregated the single-scale prediction results, validation on a truly multi-scale dataset comprising patches at distinct magnification levels remains an open objective. Although Histo-FM-based models successfully discriminated many morphological subtypes along the BE progression spectrum, diagnostic discrimination remained limited at morphologically transitional stages across the full BE spectrum, motivating the prospective investigation of a pathologist-in-the-loop computational pathology approach.

## Methods

### Dataset

This study used a dataset derived from the previously established German multicenter BarrettNet cohort^19^, which was curated for the downstream analysis. In the BarrettNet cohort, histopathological diagnoses were established within the Institute of Pathology’s accredited diagnostic workflow at the Technical University of Munich. Diagnostic assessment was performed by board-certified pathologists, including at least three expert gastrointestinal pathologists. All cases with Barrett-associated dysplasia, esophageal adenocarcinoma, and diagnostically challenging findings, including regenerative atypia and diagnostically equivocal lesions, underwent independent review by two pathologists. Cases with diagnostic disagreement were referred to an external expert gastrointestinal pathologist for adjudication. The final consensus diagnosis served as the reference standard.

In the current study, data curation and harmonization included restructuring the dataset into an analysis-ready format. Barcode reconciliation and slide-to-metadata mapping were performed to ensure reliable WSI retrieval. To verify metadata consistency, manual verification was performed against the pathology information system and associated cohort metadata. Heterogeneous metadata entries were manually standardized before model development. No diagnostic labels or clinicopathological outcomes were statistically imputed. Furthermore, non-H&E-stained slides (e.g., PAS-stained slides) and incomplete data (e.g., missing associated metadata or missing diagnosis) were excluded from downstream analyses. Slides were digitized using Leica Aperio GT 450 DX scanners at 40× magnification and 0.26 µm/pixel resolution at the Technical University of Munich.

**Fig. 7:**
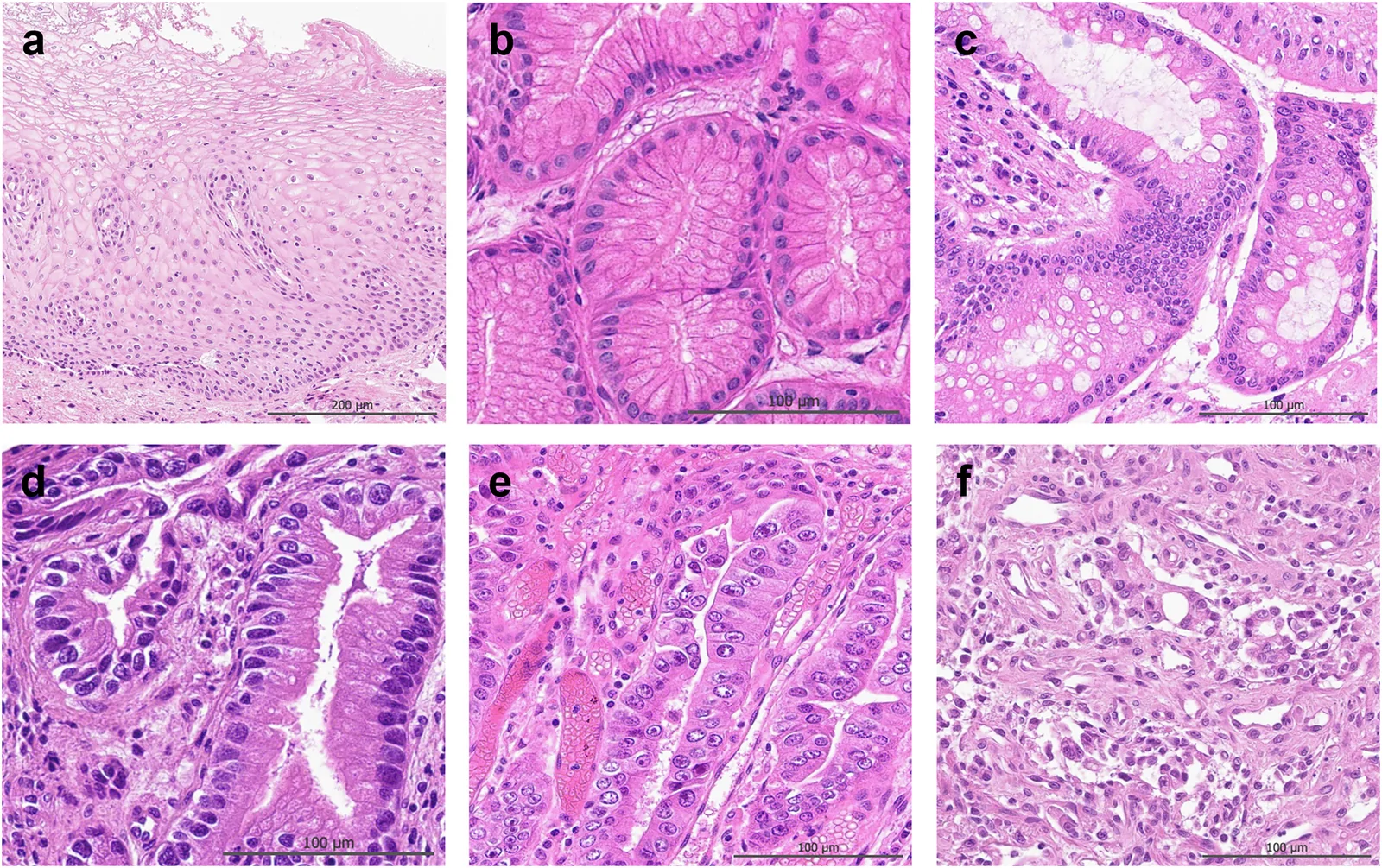
Examples of typical H&E-stained tissue subtypes. **a** Squamous mucosa; **b** Cardiac mucosa; **c:** Non-dysplastic Barrett’s mucosa; **d** Low-grade dysplastic Barrett’s; **e** High-grade dysplastic Barrett’s; **f** Esophageal adenocarcinoma (EAC). Patch **a** was taken at 10× magnification, while the remaining patches were taken at 20× magnification.

Using the cohort reference diagnosis, we formulated two clinically relevant slide-level classification tasks. Morphological subtyping denotes the differentiation of (1) non-Barrett’s normal tissue (squamous epithelium or cardiac mucosa), (2) Barrett’s metaplasia without dysplasia, (3) Barrett’s metaplasia with dysplasia (LGD and HGD), and (4) adenocarcinoma morphologies, capturing both tissue type variation and stages of neoplastic progression (Fig. 7). However, EAC detection was formulated as a binary classification task, treating adenocarcinoma as the cancer class and the remaining classes as non-cancer.

This study was conducted in accordance with the Declaration of Helsinki and approved by the Technical University of Munich Ethics Committee (approval no. 2023-665-S-CB). The need for informed consent was waived because all data were collected as part of the BarrettNet study earlier^19^ and de-identified for this study.

### WSI preprocessing

**Fig. 8:**
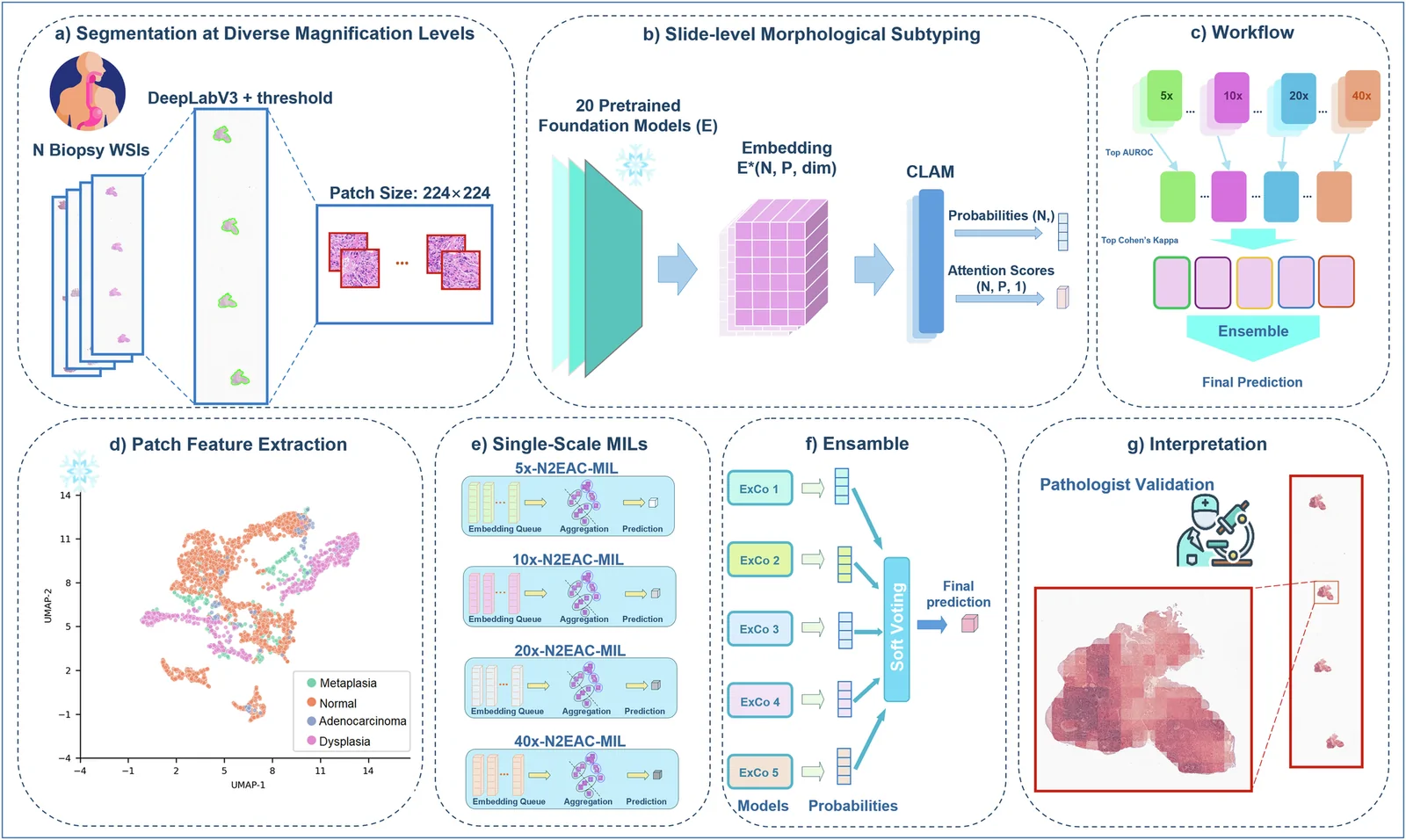
Overview of our pipeline. After slide digitalization, **a** N number of WSIs were segmented and patched into smaller tiles (every WSI has a different number P of tiles) at four different magnification levels; **b** at each magnification setting, all patches corresponding to each WSI were independently processed by each of the pretrained FMs for feature extraction, then the extracted embeddings were passed into the N2EAC-MIL classifier for the subtyping and EAC detection tasks at the slide-level manner. The output is the class probability for each WSI and additionally the patch-level attention scores; **c** Model selection workflow designed in this study to benchmark FMs. In the first round, models were selected based on their AUROC scores. In the second round, the top five models were chosen based on Cohen’s Kappa value (ExCo models); **d** Twenty encoders were utilized for patch-level feature extraction, with embedding dimensions differing from encoder to encoder. An example of the distribution of features extracted from one of the encoders (Virchow2) is shown here. The dimensionality of patch-level features for four WSIs (each from one morphological subtype) was reduced via PCA and illustrated using UMAP; **e** all WSIs were preprocessed at different magnification levels (5×, 10×, 20×, and 40×) fed as an input into different single-scale MILs; **f** probabilities obtained from the top five ExCo models at multiple magnifications were fused; **g** expert assessment was conducted based on interpreting the attention heatmap generated using the patches’ attention scores.

The process outlined in Fig. 8a begins by segmenting individual regions of interest within each WSI, distinguishing tissue areas from the background^53^. This distinction is achieved by employing the DeepLabV3-ResNet50 backbone^54^, a neural network architecture optimized for histopathological semantic segmentation. The segmentation model was refined through fine-tuning to accurately separate foreground vs. background, applying a SoftMax activation function and a confidence threshold^53^. Some predicted foreground masks and tissue regions were validated visually to ensure satisfactory segmentation.

Although most FMs have been trained at a 20× magnification level, the impact of magnification level on training models at a specific magnification level or mixing multiple magnifications for diagnostic tasks is unanswered^3^. To evaluate the impact of magnification-dependent training on the performance of BE-related diagnostic tasks, we segmented the WSIs at four magnification levels (5×, 10×, 20×, and 40×) to train single-scale N2EAC-MIL models. From segmented WSIs, the tissue foreground was split into non-overlapping 224×224-pixel tiles. All foreground patches were preserved to capture pattern heterogeneity across distinct regions and to avoid potential bias introduced by discarding foreground tissue.

### Foundation models

In this study, the benchmark included diverse FMs spanning SSL, multimodal, and large-scale histopathology-specific pretraining paradigms. Histo-FM models such as UNI/UNI2-h^1^, Virchow^55^, Virchow2^56^, CONCH^57^, CONCHv1.5^57^, Phikon^58^, Phikon-v2^58^, CTransPath^59^, Prov-Gigapath^60^, H-Optimus-0^61^, Kaiko-ViT^62^, Hibou-L^63^, Lunit-ViT-S8^44^, and MUSK^64^ were included. With varying pretraining paradigms, all these Histo-FMs adopt heterogeneous representation-learning approaches to learn histopathological feature embeddings. We also incorporated ResNet50^65^, which is a long-standing backbone in clinicopathology applications, trained on ImageNet^66^, a diverse dataset of natural images. Table 3 provides an overview of these FMs, summarizing key architectural and pretraining characteristics.

**Table 3:** Details of All FMs Included in the Study

Foundation model	Method/strategy	Embedding dimension	Framework	Architecture/scale	Params [M]	Training Mag. Level	Training dataset	No. of histopathology images	Staining type	GI Tissues	Biopsy
UNI^1^	Self distillation-based SSL	1024	DINOv2	ViT-L/16	303	20×	Private, Public	100 K WSIs	H&E	Yes	Unknown
UNI2-h^1^	Self-distillation + masked image modeling	1536	DINOv2	ViT-H/14	681	unknown	Private	350 K WSIs	H&E and IHC	Yes	Unknown
Virchow^55^	Student-teacher SSL paradigm	2560	DINOv2	ViT-H/14	632	20×	Private	1.5 M WSIs	H&E	Yes	Yes
Virchow2^56^	Student-teacher SSL with domain-specific augmentation	2560	DINOv2	ViT-H/14	632	5×, 10×, 20×, 40×	Private	3.1 M WSIs	H&E and IHC	Yes	Yes
CONCH^57^	Vision-language contrastive learning	512	iBOT	ViT-B/16	90	20×	Public, Private	1.17 M image-caption pairs	H&E-others	Yes	Yes
CONCHv1.5^57^	Vision-language contrastive learning	768	iBOT	ViT-L/16	307	20×	Public, Private	1.17 M image-caption pairs	H&E-others	Yes	Yes
Phikon^58^	Masked Image Modeling (MIM)	768	iBOT	ViT-B/16	86	20×	Public	43.37 M patches	H&E	Yes	Unknown
Phikon-v2^72^	Self distillation + MIM	1024	DINOv2	ViT-L/16	307	20×	Public	456.06 M patches	H&E	Yes	Unknown
Prov-Gigapath^60^	Masked Autoencoding	1536	DINOv2	ViT-G/14	1135	20×	Private/ Proprietary	171 K WSIs	H&E and IHC	Yes	Yes
CTransPath^59^	Semantically-relevant contrastive learning (SRCL)	768	CNN + Swin Transformer	Swin-T/14	28.3	20×	Public	15 M patches	H&E	Yes	Unknown
H-Optimus-0^61^	SSL	1536	DINOv2/iBOT	ViT-G/14	1135	20×	Private/ Proprietary	500 K WSIs	H&E	Yes	Unknown
Kaiko-ViT-S8^62^	SSL	384	DINO	ViT-S/8	22	5×, 10×, 20×, 40×	Public	29 K WSIs	H&E	Yes	Yes
Kaiko-ViT-S16^62^	SSL	384	DINO	ViT-S/16	22	5×, 10×, 20×, 40×	Public	29 K WSIs	H&E	Yes	Yes
Hibou-L^63^	SSL	1024	DINOv2	ViT-L/14	307	Unknown	Private	1.2 B patches	H&E, Cytology, Non-H&E	Yes	Veterinary biopsies
Kaiko-ViT-B8^62^	SSL	768	DINO	ViT-B/8	86	5×, 10×, 20×, 40×	Public	29 K WSIs	H&E	Yes	Yes
Kaiko-ViT-B16^62^	SSL	768	DINO	ViT-B/16	86	5×, 10×, 20×, 40×	Public	29 K WSIs	H&E	Yes	Yes
Kaiko-ViT-L14^62^	SSL	1024	DINOv2	ViT-L/14	304	5×, 10×, 20×, 40×	Public	29 K WSIs	H&E	Yes	Yes
Lunit-ViT-S8^44^	SSL	384	DINO	ViT-S/8	22	20×, 40×	Private, Public	36 K WSIs	H&E	Unknown	Unknown
MUSK^64^	Vision-language model	1024	BEiT3-inspired multimodal transformer	L/16	675	10×, 20×, 40×	Public	50 M WSIs	H&E	Yes	Unknown
ResNet50^65^	Supervised ImageNet pretraining	1024	ResNet-50	N/A	8.54	-	ImageNet-1K	N/A	N/A	N/A	N/A

### Model training and evaluation

We considered morphological subtyping and EAC detection as separate slide-level classification tasks. For model training, we employed a weakly supervised learning approach, namely MIL^35^. In this setting, groups of corresponding patches of a WSI are typically referred to as “bags”, and specific patches are referred to as “instances”^67^. Therefore, MIL enables training without fully annotated data and has become the backbone of several computational pathology projects in recent years^30,67–70^. For each FM, including Histo-FMs and ResNet50, no encoder fine-tuning was done. (Fig. 8b). All single-scale N2EAC-MIL models were trained at different magnification levels (5×, 10×, 20×, and 40×) for each subtyping task and cancer detection (Fig. 8b). We employed a 10-fold Monte Carlo cross-validation, with 80% of the data assigned to the training set, 10% to the validation set, and 10% to the test set in each fold. Patient-level stratification prevented data leakage, and weighted sampling addressed class imbalance during training.

We comprehensively and systematically examined model performance across maximum epochs (1, 2, 5, 10, 20, and 50) to determine the upper bound of performance throughout training. Early stopping with patience was applied for longer training schedules to prevent overfitting. AUROC was evaluated to select the top-performing models (Fig. 8c). Each diagnostic category exhibits substantial morphological heterogeneity, leading to distinct distributions of learned feature representations (Fig. 8d). Single-scale models trained at a single magnification level enabled us to observe how the magnification level influences model performance (Fig. 8e).

Following intensive N2EAC-MIL hyperparameter optimization with different optimizers, learning rates, and regularization schemes, attention networks, bag loss, and model types were evaluated. CLAM with a single branch of the attention network was used. We expanded the original model architecture by increasing the feature projection and attention hidden dimensions to 768 and 512, thereby enhancing the network’s capacity to potentially capture more complex histopathological patterns. This modification was based on improvements observed during training experiments, resulting in better performance on our morphological subtyping and EAC detection tasks.

Moreover, the model was trained with a learning rate of 1e-5, regularization of 1e-7, and a bag weight of 0.7, using the Adam optimizer^71^ as the optimization method; a bag-level cross-entropy loss was also employed to optimize the predictions. Additionally, we applied dropout with a rate of 0.25 to enhance generalization. To improve the performance and learning of patch-level importance weights, we expanded the attention network’s hidden layer to 512 dimensions, thereby strengthening the network’s capacity. Moreover, during training, the bag-level class assignment is based on an attention-weighted aggregation over the entire bag. Class imbalance was addressed by incorporating weighted sampling. All experiments were trained using a single GPU (GeForce RTX 3090).

Cohen’s kappa was used to assess agreement between model predictions and the reference standard. Multirater agreement was assessed to estimate consistency among all raters involved in the study, including single-scale models and the reference standard for each task. Fleiss’ kappa quantifies the consistency of model predictions, providing a measure of reliability across various backbones. Moreover, pairwise agreement was assessed between each model and the reference standard, as well as between pairs of models. In both multi-rater and pairwise agreement assessments, a fold-wise kappa calculation has been conducted. All results are reported as mean ± standard deviation and 95% confidence interval to illustrate variability and the precision of the estimated mean.

### Ensembling

A probability-based soft voting ensemble approach was applied to refine our final prediction in both tasks, including morphological subtyping and cancer detection, whereby the final prediction probabilities from the top five single-scale N2EAC-MIL models are averaged. The top five models, identified by Cohen’s kappa, were selected for soft voting ensembling (Fig. 8f). To investigate whether the ensemble improves performance, we employed the one-sided Wilcoxon signed-rank test, performed separately for each metric across 10-fold Monte Carlo cross-validation runs. Therefore, the Wilcoxon tests were used to assess the directional improvement in this study. The significance level considered is 0.05. We hypothesize that ensembling can improve performance for morphological subtyping and cancer detection across single-scale models and performance metrics.

### Pathologist validation

We assessed the model’s outputs through expert review of attention heatmaps to examine whether the model’s attention aligns with diagnostically informative morphology (Fig. 8g). The primary objective was to assess whether the model’s attention was directed toward diagnostically relevant morphological regions, thereby evaluating the morphological alignment of the model’s prediction heatmaps. We randomly selected one WSI from each morphological subtype correctly predicted by the top five ExCo models and generated heatmaps at the magnification level at which each model performed best (Table 1). To compare models’ outputs for spatial correspondence with diagnostically relevant tissue, a board-certified pathologist who participated in the original diagnostic assessment of the cohort evaluated the heatmaps. The reviewer assessed the heatmaps and assigned a score using a five-point Likert scale from 1 (least alignment) to 5 (highest alignment) to evaluate the alignment of the model’s output with diagnostically relevant tissue. In addition, the reviewer provided narrative feedback on the morphological relevance of the highlighted regions for each WSI.

As dysplasia is the most diagnostically challenging subtype with heterogeneous morphology, we assessed whether attention heatmaps aligned with histomorphologically relevant tissue regions in the dysplasia and EAC cases, regardless of classification outcome. To extend the expert evaluation and strengthen the heatmap assessment, a qualitative and semi-quantitative localization assessment was conducted. As a secondary aim, to quantitatively assess inter-rater reliability, the intraclass correlation coefficient (ICC) was computed. We randomly selected a subset of heatmaps, comprising 10% of dysplastic and adenocarcinoma slides, derived from ExCo models for morphological subtyping. We selected slides regardless of the prediction outcome (success/failure) and the model’s attention pattern. Three pathologists (one board-certified pathologist, one experienced pathology resident, and a junior pathology resident) independently reviewed the heatmaps of BE dysplastic and adenocarcinoma tissue, with access to the original WSIs and their corresponding pathology reports. Prior to assessment, pathologists received standardized instructions and information on heatmap visualization and the interpretation protocol. To minimize assessment bias, attention maps were presented without the model identity or prediction. A score was assigned to each heatmap on a 5-point Likert scale to assess whether the attention map aligned with diagnostically relevant regions. Narrative feedback on the heatmaps was received from the reviewers to support the interpretation of the heatmap findings.

### Reporting summary

Further information on research design is available in the Nature Research Reporting Summary linked to this article.

## Supplementary information

Supplementary information

Reporting Summary

## Data Availability

The datasets generated and/or analyzed during the current study are not publicly available due to legal, patient privacy, and institutional data-sharing restrictions, but are available upon reasonable request, subject to approval by the data-owning institutions and applicable ethical, legal, and data protection requirements.

## Code availability

The code used for feature extraction and model training, as described in the Methods section, was obtained from the public repositories of the TRIDENT and CLAM frameworks and pretrained foundation models. Experiments and analysis were performed using Python (version 3.11.7), the OpenSlide package (version 1.3.1), and the PyTorch package (version 2.5.1+cu124). Details of model architectures, training procedures, and evaluation parameters are provided in the Methods section. Custom Python scripts used for model evaluation, ensemble generation, statistical analysis, and figure generation are available from the corresponding author upon reasonable request.
