# Supplementary material for: Towards reliable morphological subtyping and cancer detection on Barrett’s esophagus pathology through ensembling foundation models
Source: NPJ Digit Med. 2026 Oct 21;9:811. doi: 10.1038/s41746-026-03329-3 (PMC13694699; doi:10.1038/s41746-026-03329-3)
Supplement: Supplementary file 1 [file 41746_2026_3329_MOESM1_ESM.pdf]

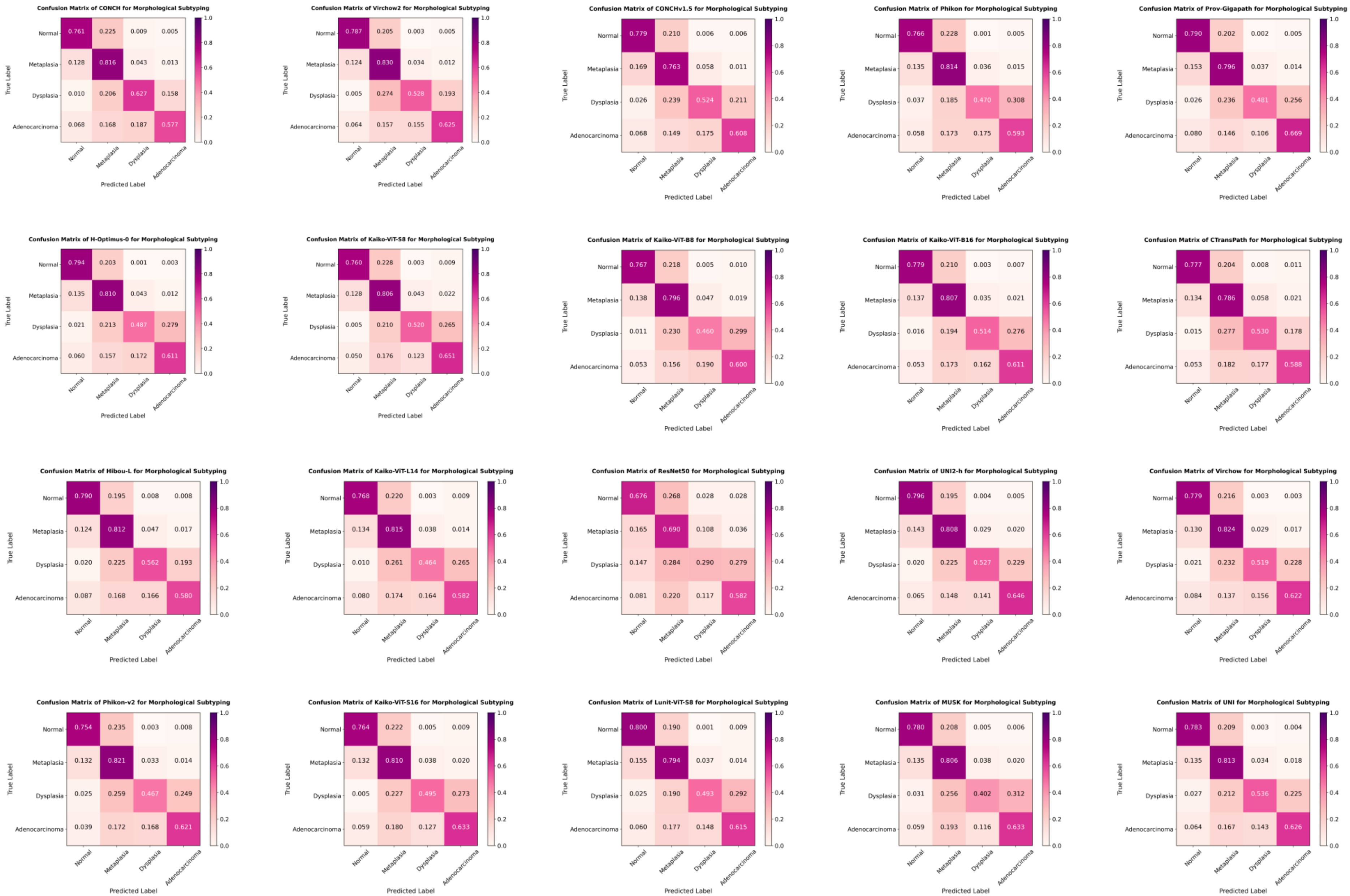

**Figure S1 | Confusion Matrices of Models for Morphological Subtyping.**

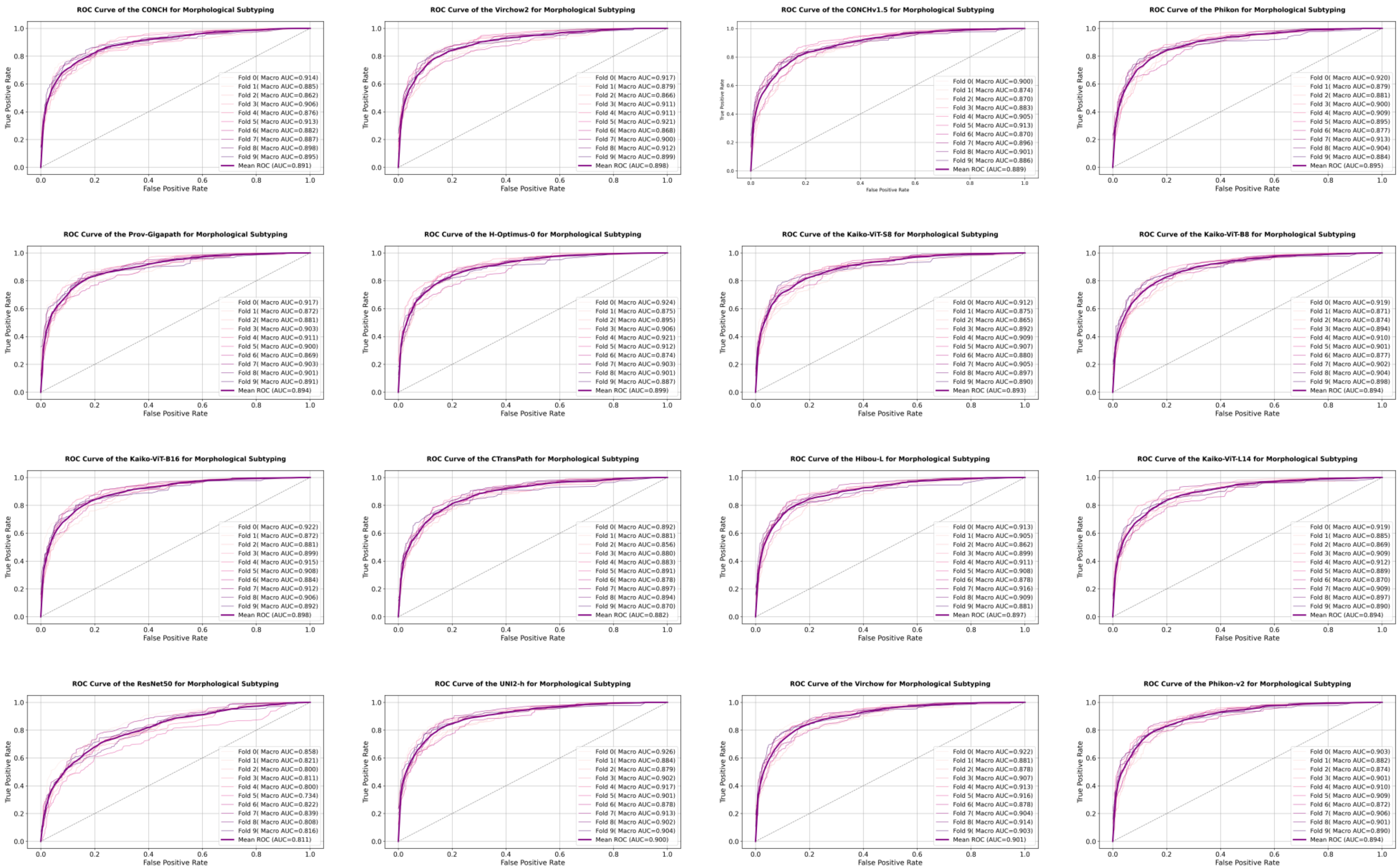
ROC Curve of the CONCH for Morphological Subtyping
True Positive Rate
False Positive Rate
Fold 0( Macro AUC=0.914)
Fold 1( Macro AUC=0.885)
Fold 2( Macro AUC=0.862)
Fold 3( Macro AUC=0.906)
Fold 4( Macro AUC=0.876)
Fold 5( Macro AUC=0.913)
Fold 6( Macro AUC=0.882)
Fold 7( Macro AUC=0.887)
Fold 8( Macro AUC=0.898)
Fold 9( Macro AUC=0.895)
Mean ROC (AUC=0.891)
ROC Curve of the Virchow2 for Morphological Subtyping
Fold 0( Macro AUC=0.917)
Fold 1( Macro AUC=0.879)
Fold 2( Macro AUC=0.866)
Fold 3( Macro AUC=0.911)
Fold 4( Macro AUC=0.911)
Fold 5( Macro AUC=0.921)
Fold 6( Macro AUC=0.868)
Fold 7( Macro AUC=0.900)
Fold 8( Macro AUC=0.912)
Fold 9( Macro AUC=0.899)
Mean ROC (AUC=0.898)
ROC Curve of the CONCHv1.5 for Morphological Subtyping
Fold 0( Macro AUC=0.900)
Fold 1( Macro AUC=0.874)
Fold 2( Macro AUC=0.870)
Fold 3( Macro AUC=0.883)
Fold 4( Macro AUC=0.905)
Fold 5( Macro AUC=0.913)
Fold 6( Macro AUC=0.870)
Fold 7( Macro AUC=0.896)
Fold 8( Macro AUC=0.901)
Fold 9( Macro AUC=0.886)
Mean ROC (AUC=0.889)
ROC Curve of the Phikon for Morphological Subtyping
Fold 0( Macro AUC=0.920)
Fold 1( Macro AUC=0.879)
Fold 2( Macro AUC=0.881)
Fold 3( Macro AUC=0.900)
Fold 4( Macro AUC=0.909)
Fold 5( Macro AUC=0.895)
Fold 6( Macro AUC=0.877)
Fold 7( Macro AUC=0.913)
Fold 8( Macro AUC=0.904)
Fold 9( Macro AUC=0.884)
Mean ROC (AUC=0.895)
ROC Curve of the Prov-Gigapath for Morphological Subtyping
Fold 0( Macro AUC=0.917)
Fold 1( Macro AUC=0.872)
Fold 2( Macro AUC=0.881)
Fold 3( Macro AUC=0.903)
Fold 4( Macro AUC=0.911)
Fold 5( Macro AUC=0.900)
Fold 6( Macro AUC=0.869)
Fold 7( Macro AUC=0.903)
Fold 8( Macro AUC=0.901)
Fold 9( Macro AUC=0.891)
Mean ROC (AUC=0.894)
ROC Curve of the H-Optimus-0 for Morphological Subtyping
Fold 0( Macro AUC=0.924)
Fold 1( Macro AUC=0.875)
Fold 2( Macro AUC=0.895)
Fold 3( Macro AUC=0.906)
Fold 4( Macro AUC=0.921)
Fold 5( Macro AUC=0.912)
Fold 6( Macro AUC=0.874)
Fold 7( Macro AUC=0.903)
Fold 8( Macro AUC=0.901)
Fold 9( Macro AUC=0.887)
Mean ROC (AUC=0.899)
ROC Curve of the Kaiko-ViT-S8 for Morphological Subtyping
Fold 0( Macro AUC=0.912)
Fold 1( Macro AUC=0.875)
Fold 2( Macro AUC=0.865)
Fold 3( Macro AUC=0.892)
Fold 4( Macro AUC=0.909)
Fold 5( Macro AUC=0.907)
Fold 6( Macro AUC=0.880)
Fold 7( Macro AUC=0.905)
Fold 8( Macro AUC=0.897)
Fold 9( Macro AUC=0.890)
Mean ROC (AUC=0.893)
ROC Curve of the Kaiko-ViT-B8 for Morphological Subtyping
Fold 0( Macro AUC=0.919)
Fold 1( Macro AUC=0.871)
Fold 2( Macro AUC=0.874)
Fold 3( Macro AUC=0.894)
Fold 4( Macro AUC=0.910)
Fold 5( Macro AUC=0.901)
Fold 6( Macro AUC=0.877)
Fold 7( Macro AUC=0.902)
Fold 8( Macro AUC=0.904)
Fold 9( Macro AUC=0.898)
Mean ROC (AUC=0.894)
ROC Curve of the Kaiko-ViT-B16 for Morphological Subtyping
Fold 0( Macro AUC=0.922)
Fold 1( Macro AUC=0.872)
Fold 2( Macro AUC=0.881)
Fold 3( Macro AUC=0.899)
Fold 4( Macro AUC=0.915)
Fold 5( Macro AUC=0.908)
Fold 6( Macro AUC=0.884)
Fold 7( Macro AUC=0.912)
Fold 8( Macro AUC=0.906)
Fold 9( Macro AUC=0.892)
Mean ROC (AUC=0.898)
ROC Curve of the CTransPath for Morphological Subtyping
Fold 0( Macro AUC=0.892)
Fold 1( Macro AUC=0.881)
Fold 2( Macro AUC=0.856)
Fold 3( Macro AUC=0.880)
Fold 4( Macro AUC=0.883)
Fold 5( Macro AUC=0.891)
Fold 6( Macro AUC=0.878)
Fold 7( Macro AUC=0.897)
Fold 8( Macro AUC=0.894)
Fold 9( Macro AUC=0.870)
Mean ROC (AUC=0.882)
ROC Curve of the Hibou-L for Morphological Subtyping
Fold 0( Macro AUC=0.913)
Fold 1( Macro AUC=0.905)
Fold 2( Macro AUC=0.862)
Fold 3( Macro AUC=0.899)
Fold 4( Macro AUC=0.911)
Fold 5( Macro AUC=0.908)
Fold 6( Macro AUC=0.878)
Fold 7( Macro AUC=0.916)
Fold 8( Macro AUC=0.909)
Fold 9( Macro AUC=0.881)
Mean ROC (AUC=0.897)
ROC Curve of the Kaiko-ViT-L14 for Morphological Subtyping
Fold 0( Macro AUC=0.919)
Fold 1( Macro AUC=0.885)
Fold 2( Macro AUC=0.869)
Fold 3( Macro AUC=0.909)
Fold 4( Macro AUC=0.912)
Fold 5( Macro AUC=0.889)
Fold 6( Macro AUC=0.870)
Fold 7( Macro AUC=0.909)
Fold 8( Macro AUC=0.897)
Fold 9( Macro AUC=0.890)
Mean ROC (AUC=0.894)
ROC Curve of the ResNet50 for Morphological Subtyping
Fold 0( Macro AUC=0.858)
Fold 1( Macro AUC=0.821)
Fold 2( Macro AUC=0.800)
Fold 3( Macro AUC=0.811)
Fold 4( Macro AUC=0.800)
Fold 5( Macro AUC=0.734)
Fold 6( Macro AUC=0.822)
Fold 7( Macro AUC=0.839)
Fold 8( Macro AUC=0.808)
Fold 9( Macro AUC=0.816)
Mean ROC (AUC=0.811)
ROC Curve of the UNI2-h for Morphological Subtyping
Fold 0( Macro AUC=0.926)
Fold 1( Macro AUC=0.884)
Fold 2( Macro AUC=0.879)
Fold 3( Macro AUC=0.902)
Fold 4( Macro AUC=0.917)
Fold 5( Macro AUC=0.901)
Fold 6( Macro AUC=0.878)
Fold 7( Macro AUC=0.913)
Fold 8( Macro AUC=0.902)
Fold 9( Macro AUC=0.904)
Mean ROC (AUC=0.900)
ROC Curve of the Virchow for Morphological Subtyping
Fold 0( Macro AUC=0.922)
Fold 1( Macro AUC=0.881)
Fold 2( Macro AUC=0.878)
Fold 3( Macro AUC=0.907)
Fold 4( Macro AUC=0.913)
Fold 5( Macro AUC=0.916)
Fold 6( Macro AUC=0.878)
Fold 7( Macro AUC=0.904)
Fold 8( Macro AUC=0.914)
Fold 9( Macro AUC=0.903)
Mean ROC (AUC=0.901)
ROC Curve of the Phikon-v2 for Morphological Subtyping
Fold 0( Macro AUC=0.903)
Fold 1( Macro AUC=0.882)
Fold 2( Macro AUC=0.874)
Fold 3( Macro AUC=0.901)
Fold 4( Macro AUC=0.910)
Fold 5( Macro AUC=0.909)
Fold 6( Macro AUC=0.872)
Fold 7( Macro AUC=0.906)
Fold 8( Macro AUC=0.901)
Fold 9( Macro AUC=0.890)
Mean ROC (AUC=0.894)

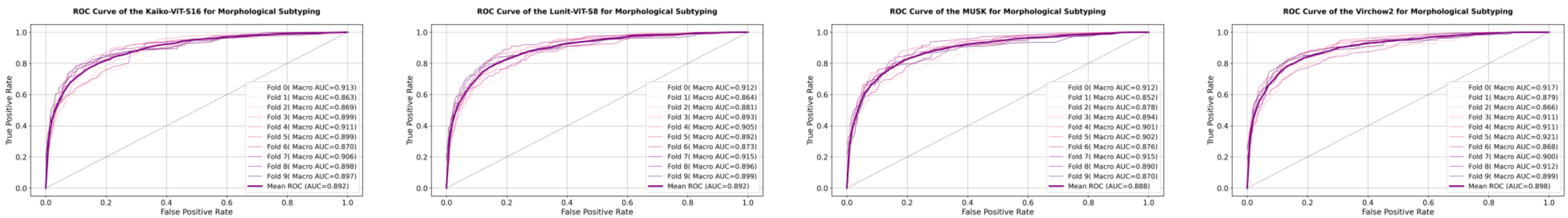


**Figure S2 | Receiver Operating Characteristic Curve of Models for Morphological Subtyping.**

**Table S1| Micro and Weighted Performance Metrics of Models for Morphological Subtyping.**

| Foundation Models | Magnification | Precision (Micro Average)<br>Recall (Micro Average)<br>Recall (Weighted Average)<br>F1-score (Micro Average)<br>Mean ± SD [95% CI Half-Width] | Precision (Weighted Average)<br>Mean ± SD [95% CI Half-Width] | Specificity (Weighted Average)<br>Mean ± SD [95% CI Half-Width] | F1-Score (Weighted Average)<br>Mean ± SD [95% CI Half-Width] |
|---|---|---|---|---|---|
| Virchow2 | 20x | 0.786 ± 0.012 [± 0.009] | 0.790 ± 0.015 [± 0.011] | 0.851 ± 0.010 [± 0.007] | 0.786 ± 0.013 [± 0.010] |
| Virchow | 20x | 0.778 ± 0.022 [± 0.016] | 0.783 ± 0.020 [± 0.014] | 0.846 ± 0.013 [± 0.009] | 0.778 ± 0.021 [± 0.015] |
| UNI2-h | 20x | 0.780 ± 0.016 [± 0.011] | 0.785 ± 0.017 [± 0.012] | 0.850 ± 0.018 [± 0.013] | 0.780 ± 0.017 [± 0.012] |
| UNI | 20x | 0.776 ± 0.019 [± 0.013] | 0.781 ± 0.021 [± 0.015] | 0.847 ± 0.012 [± 0.009] | 0.777 ± 0.019 [± 0.014] |
| ResNet50 | 20x | 0.661 ± 0.023 [± 0.016] | 0.692 ± 0.030 [± 0.022] | 0.808 ± 0.021 [± 0.015] | 0.672 ± 0.024 [± 0.017] |
| Phikon-v2 | 20x | 0.765 ± 0.018 [± 0.013] | 0.772 ± 0.022 [± 0.016] | 0.831 ± 0.022 [± 0.016] | 0.765 ± 0.020 [± 0.014] |
| Phikon | 20x | 0.763 ± 0.021 [± 0.015] | 0.771 ± 0.024 [± 0.017] | 0.837 ± 0.025 [± 0.018] | 0.762 ± 0.024 [± 0.017] |
| MUSK | 20x | 0.767 ± 0.018 [± 0.013] | 0.773 ± 0.020 [± 0.014] | 0.842 ± 0.021 [± 0.015] | 0.768 ± 0.019 [± 0.014] |
| Lunit-ViT-S8 | 20x | 0.772 ± 0.026 [± 0.018] | 0.778 ± 0.029 [± 0.021] | 0.849 ± 0.019 [± 0.014] | 0.772 ± 0.026 [± 0.019] |
| Kaiko-ViT-S8 | 20x | 0.763 ± 0.020 [± 0.015] | 0.773 ± 0.021 [± 0.015] | 0.840 ± 0.020 [± 0.014] | 0.764 ± 0.021 [± 0.015] |
| Kaiko-ViT-S16 | 20x | 0.764 ± 0.017 [± 0.012] | 0.774 ± 0.017 [± 0.012] | 0.838 ± 0.024 [± 0.017] | 0.765 ± 0.018 [± 0.013] |
| Kaiko-ViT-L14 | 5x | 0.766 ± 0.020 [± 0.014] | 0.772 ± 0.023 [± 0.016] | 0.837 ± 0.024 [± 0.017] | 0.766 ± 0.020 [± 0.014] |
| Kaiko-ViT-B8 | 20x | 0.756 ± 0.019 [± 0.014] | 0.769 ± 0.020 [± 0.014] | 0.843 ± 0.021 [± 0.015] | 0.758 ± 0.020 [± 0.014] |
| Kaiko-ViT-B16 | 20x | 0.769 ± 0.019 [± 0.013] | 0.778 ± 0.018 [± 0.013] | 0.846 ± 0.018 [± 0.013] | 0.769 ± 0.020 [± 0.014] |
| H-Optimus-0 | 20x | 0.775 ± 0.024 [± 0.017] | 0.783 ± 0.024 [± 0.017] | 0.849 ± 0.021 [± 0.015] | 0.776 ± 0.024 [± 0.017] |
| Hibou-L | 5x | 0.777 ± 0.018 [± 0.013] | 0.786 ± 0.017 [± 0.012] | 0.855 ± 0.011 [± 0.008] | 0.779 ± 0.017 [± 0.012] |
| Prov-Gigapath | 20x | 0.771 ± 0.024 [± 0.017] | 0.776 ± 0.024 [± 0.017] | 0.844 ± 0.022 [± 0.016] | 0.771 ± 0.025 [± 0.018] |
| CTransPath | 10x | 0.759 ± 0.020 [± 0.014] | 0.772 ± 0.025 [± 0.018] | 0.849 ± 0.015 [± 0.011] | 0.763 ± 0.022 [± 0.016] |
| CONCHv1.5 | 20x | 0.748 ± 0.018 [± 0.013] | 0.762 ± 0.022 [± 0.015] | 0.836 ± 0.010 [± 0.007] | 0.751 ± 0.019 [± 0.014] |
| CONCH | 10x | 0.770 ± 0.015 [± 0.011] | 0.780 ± 0.020 [± 0.015] | 0.841 ± 0.018 [± 0.013] | 0.771 ± 0.017 [± 0.012] |

SD: Standard Deviation; CI: Confidence Interval.

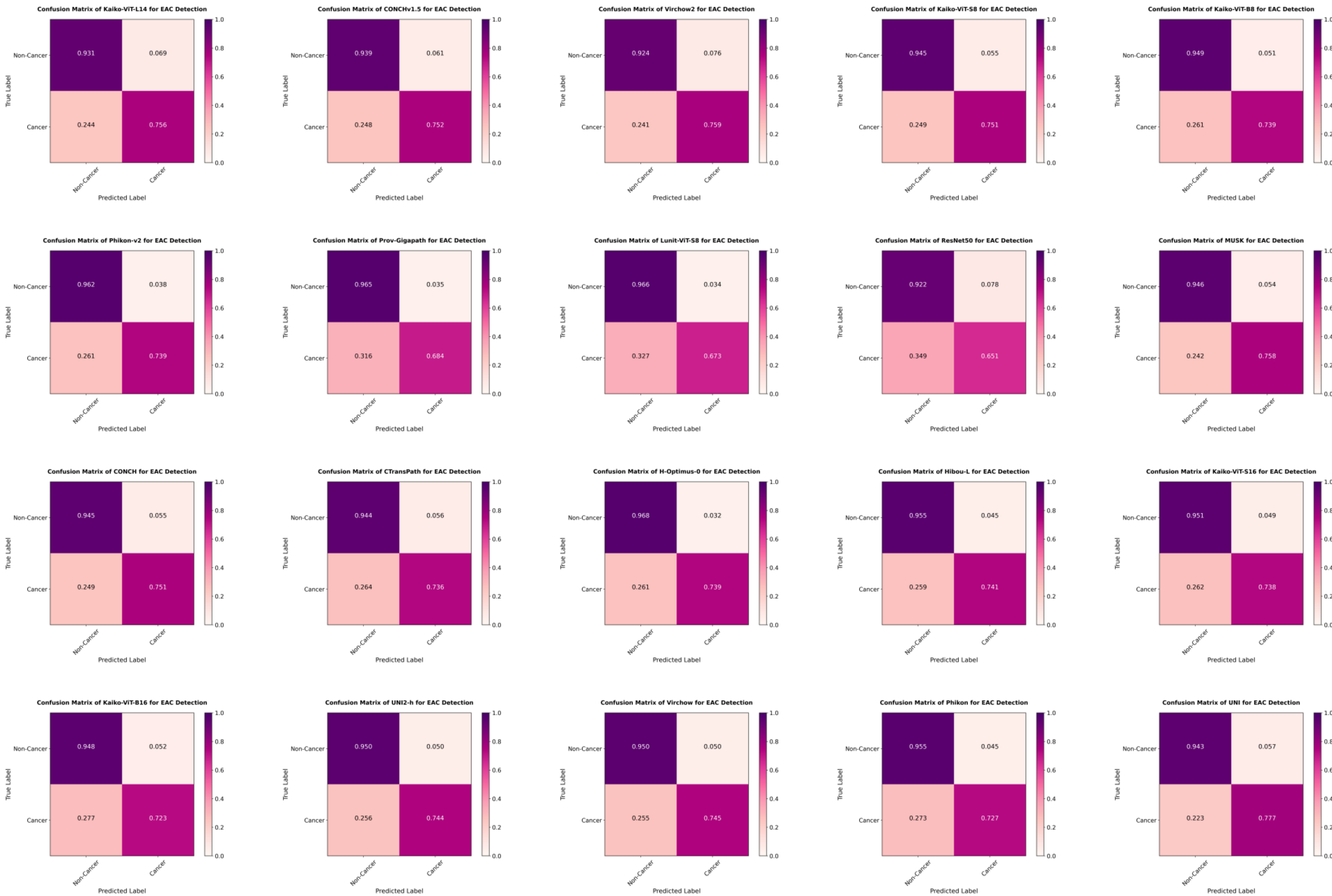


**Figure S3 |Confusion Matrices of Models for EAC Detection.**

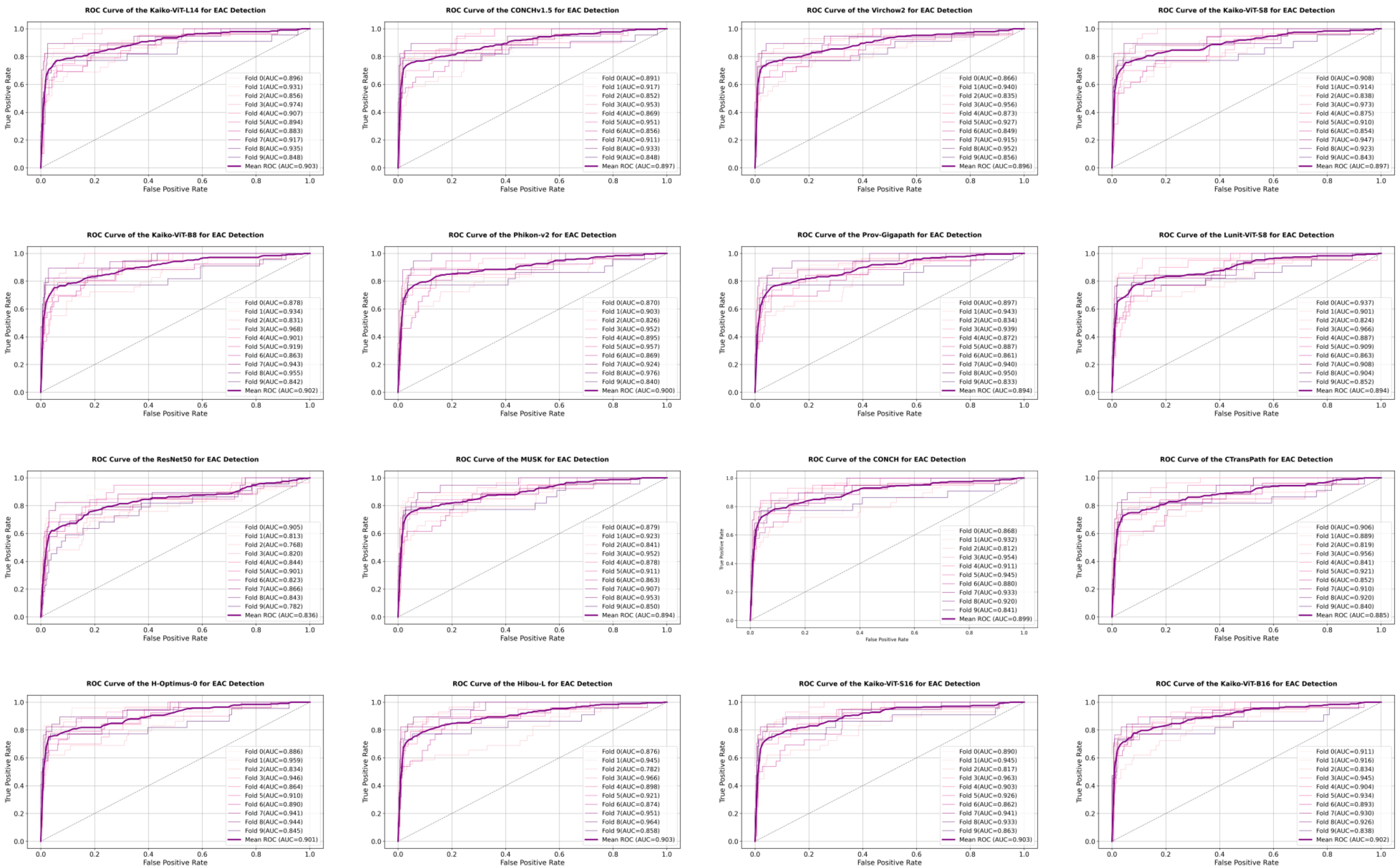
ROC Curve of the Kaiko-ViT-L14 for EAC Detection
Fold 0(AUC=0.896)
Fold 1(AUC=0.931)
Fold 2(AUC=0.856)
Fold 3(AUC=0.974)
Fold 4(AUC=0.907)
Fold 5(AUC=0.894)
Fold 6(AUC=0.883)
Fold 7(AUC=0.917)
Fold 8(AUC=0.935)
Fold 9(AUC=0.848)
Mean ROC (AUC=0.903)
True Positive Rate
False Positive Rate
ROC Curve of the CONCHv1.5 for EAC Detection
Fold 0(AUC=0.891)
Fold 1(AUC=0.917)
Fold 2(AUC=0.852)
Fold 3(AUC=0.953)
Fold 4(AUC=0.869)
Fold 5(AUC=0.951)
Fold 6(AUC=0.856)
Fold 7(AUC=0.911)
Fold 8(AUC=0.933)
Fold 9(AUC=0.848)
Mean ROC (AUC=0.897)
True Positive Rate
False Positive Rate
ROC Curve of the Virchow2 for EAC Detection
Fold 0(AUC=0.866)
Fold 1(AUC=0.940)
Fold 2(AUC=0.835)
Fold 3(AUC=0.956)
Fold 4(AUC=0.873)
Fold 5(AUC=0.927)
Fold 6(AUC=0.849)
Fold 7(AUC=0.915)
Fold 8(AUC=0.952)
Fold 9(AUC=0.856)
Mean ROC (AUC=0.896)
True Positive Rate
False Positive Rate
ROC Curve of the Kaiko-ViT-S8 for EAC Detection
Fold 0(AUC=0.908)
Fold 1(AUC=0.914)
Fold 2(AUC=0.838)
Fold 3(AUC=0.973)
Fold 4(AUC=0.875)
Fold 5(AUC=0.910)
Fold 6(AUC=0.854)
Fold 7(AUC=0.947)
Fold 8(AUC=0.923)
Fold 9(AUC=0.843)
Mean ROC (AUC=0.897)
True Positive Rate
False Positive Rate
ROC Curve of the Kaiko-ViT-B8 for EAC Detection
Fold 0(AUC=0.878)
Fold 1(AUC=0.934)
Fold 2(AUC=0.831)
Fold 3(AUC=0.968)
Fold 4(AUC=0.901)
Fold 5(AUC=0.919)
Fold 6(AUC=0.863)
Fold 7(AUC=0.943)
Fold 8(AUC=0.955)
Fold 9(AUC=0.842)
Mean ROC (AUC=0.902)
True Positive Rate
False Positive Rate
ROC Curve of the Phikon-v2 for EAC Detection
Fold 0(AUC=0.870)
Fold 1(AUC=0.903)
Fold 2(AUC=0.826)
Fold 3(AUC=0.952)
Fold 4(AUC=0.895)
Fold 5(AUC=0.957)
Fold 6(AUC=0.869)
Fold 7(AUC=0.924)
Fold 8(AUC=0.976)
Fold 9(AUC=0.840)
Mean ROC (AUC=0.900)
True Positive Rate
False Positive Rate
ROC Curve of the Prov-Gigapath for EAC Detection
Fold 0(AUC=0.897)
Fold 1(AUC=0.943)
Fold 2(AUC=0.834)
Fold 3(AUC=0.939)
Fold 4(AUC=0.872)
Fold 5(AUC=0.887)
Fold 6(AUC=0.861)
Fold 7(AUC=0.940)
Fold 8(AUC=0.950)
Fold 9(AUC=0.833)
Mean ROC (AUC=0.894)
True Positive Rate
False Positive Rate
ROC Curve of the Lunit-ViT-S8 for EAC Detection
Fold 0(AUC=0.937)
Fold 1(AUC=0.901)
Fold 2(AUC=0.824)
Fold 3(AUC=0.966)
Fold 4(AUC=0.887)
Fold 5(AUC=0.909)
Fold 6(AUC=0.863)
Fold 7(AUC=0.908)
Fold 8(AUC=0.904)
Fold 9(AUC=0.852)
Mean ROC (AUC=0.894)
True Positive Rate
False Positive Rate
ROC Curve of the ResNet50 for EAC Detection
Fold 0(AUC=0.905)
Fold 1(AUC=0.813)
Fold 2(AUC=0.768)
Fold 3(AUC=0.820)
Fold 4(AUC=0.844)
Fold 5(AUC=0.901)
Fold 6(AUC=0.823)
Fold 7(AUC=0.866)
Fold 8(AUC=0.843)
Fold 9(AUC=0.782)
Mean ROC (AUC=0.836)
True Positive Rate
False Positive Rate
ROC Curve of the MUSK for EAC Detection
Fold 0(AUC=0.879)
Fold 1(AUC=0.923)
Fold 2(AUC=0.841)
Fold 3(AUC=0.952)
Fold 4(AUC=0.878)
Fold 5(AUC=0.911)
Fold 6(AUC=0.863)
Fold 7(AUC=0.907)
Fold 8(AUC=0.953)
Fold 9(AUC=0.850)
Mean ROC (AUC=0.894)
True Positive Rate
False Positive Rate
ROC Curve of the CONCH for EAC Detection
Fold 0(AUC=0.868)
Fold 1(AUC=0.932)
Fold 2(AUC=0.812)
Fold 3(AUC=0.954)
Fold 4(AUC=0.911)
Fold 5(AUC=0.945)
Fold 6(AUC=0.880)
Fold 7(AUC=0.933)
Fold 8(AUC=0.920)
Fold 9(AUC=0.841)
Mean ROC (AUC=0.899)
True Positive Rate
False Positive Rate
ROC Curve of the CTransPath for EAC Detection
Fold 0(AUC=0.906)
Fold 1(AUC=0.889)
Fold 2(AUC=0.819)
Fold 3(AUC=0.956)
Fold 4(AUC=0.841)
Fold 5(AUC=0.921)
Fold 6(AUC=0.852)
Fold 7(AUC=0.910)
Fold 8(AUC=0.920)
Fold 9(AUC=0.840)
Mean ROC (AUC=0.885)
True Positive Rate
False Positive Rate
ROC Curve of the H-Optimus-0 for EAC Detection
Fold 0(AUC=0.886)
Fold 1(AUC=0.959)
Fold 2(AUC=0.834)
Fold 3(AUC=0.946)
Fold 4(AUC=0.864)
Fold 5(AUC=0.910)
Fold 6(AUC=0.890)
Fold 7(AUC=0.941)
Fold 8(AUC=0.944)
Fold 9(AUC=0.845)
Mean ROC (AUC=0.901)
True Positive Rate
False Positive Rate
ROC Curve of the Hibou-L for EAC Detection
Fold 0(AUC=0.876)
Fold 1(AUC=0.945)
Fold 2(AUC=0.782)
Fold 3(AUC=0.966)
Fold 4(AUC=0.898)
Fold 5(AUC=0.921)
Fold 6(AUC=0.874)
Fold 7(AUC=0.951)
Fold 8(AUC=0.964)
Fold 9(AUC=0.858)
Mean ROC (AUC=0.903)
True Positive Rate
False Positive Rate
ROC Curve of the Kaiko-ViT-S16 for EAC Detection
Fold 0(AUC=0.890)
Fold 1(AUC=0.945)
Fold 2(AUC=0.817)
Fold 3(AUC=0.963)
Fold 4(AUC=0.903)
Fold 5(AUC=0.926)
Fold 6(AUC=0.862)
Fold 7(AUC=0.941)
Fold 8(AUC=0.933)
Fold 9(AUC=0.863)
Mean ROC (AUC=0.903)
True Positive Rate
False Positive Rate
ROC Curve of the Kaiko-ViT-B16 for EAC Detection
Fold 0(AUC=0.911)
Fold 1(AUC=0.916)
Fold 2(AUC=0.834)
Fold 3(AUC=0.945)
Fold 4(AUC=0.904)
Fold 5(AUC=0.934)
Fold 6(AUC=0.893)
Fold 7(AUC=0.930)
Fold 8(AUC=0.926)
Fold 9(AUC=0.838)
Mean ROC (AUC=0.902)
True Positive Rate
False Positive Rate

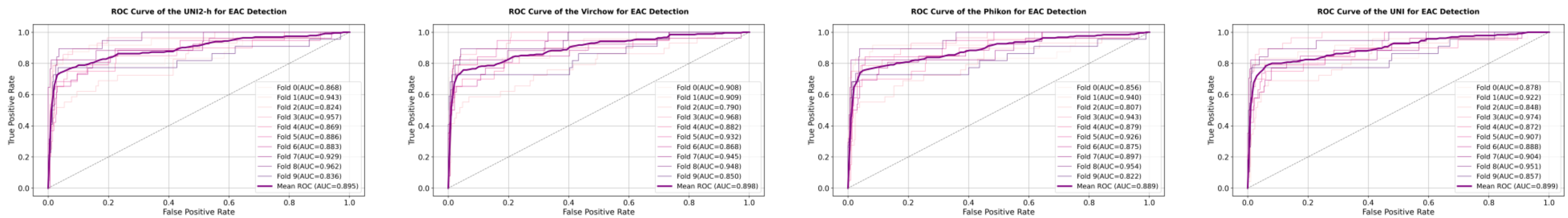


**Figure S4 |Receiver Operating Characteristic Curve of Models for EAC Detection.**

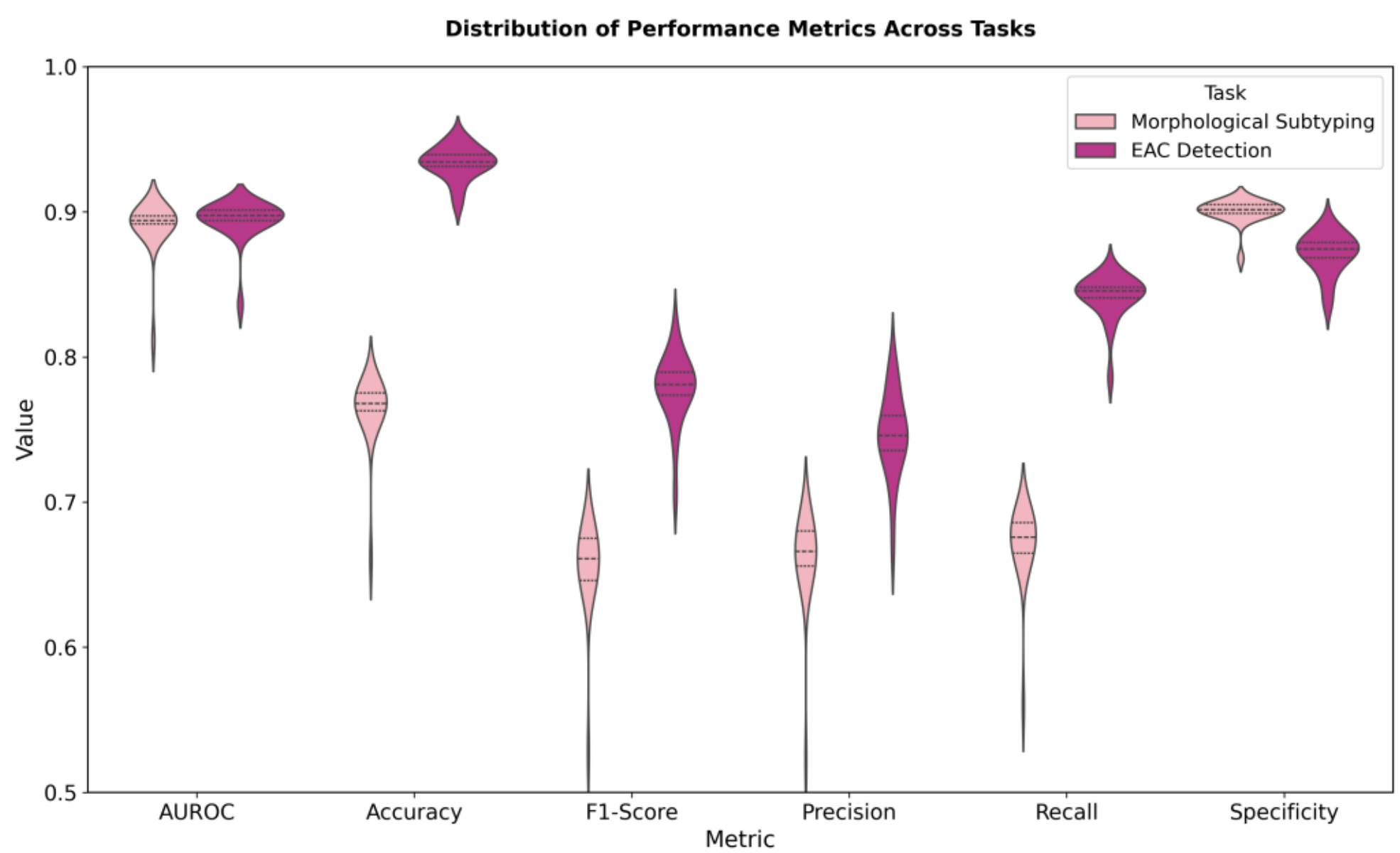


**Figure S5 | Distribution of N2EAC-MILs' Performance Metrics for Each Task Across All Foundation Models.**

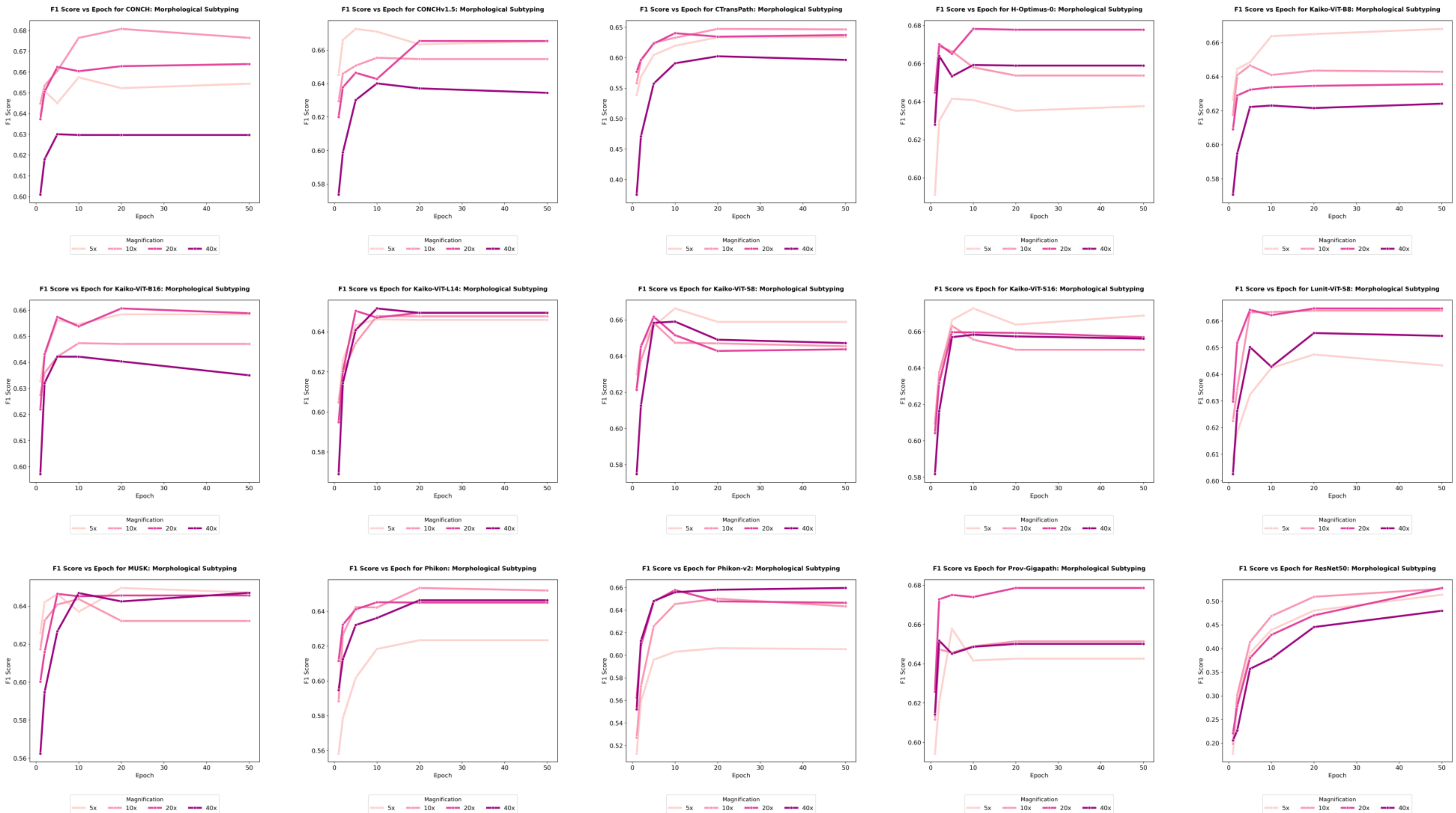


**Figure S6 | Models' F1 Score Trend for Morphological Subtyping.**

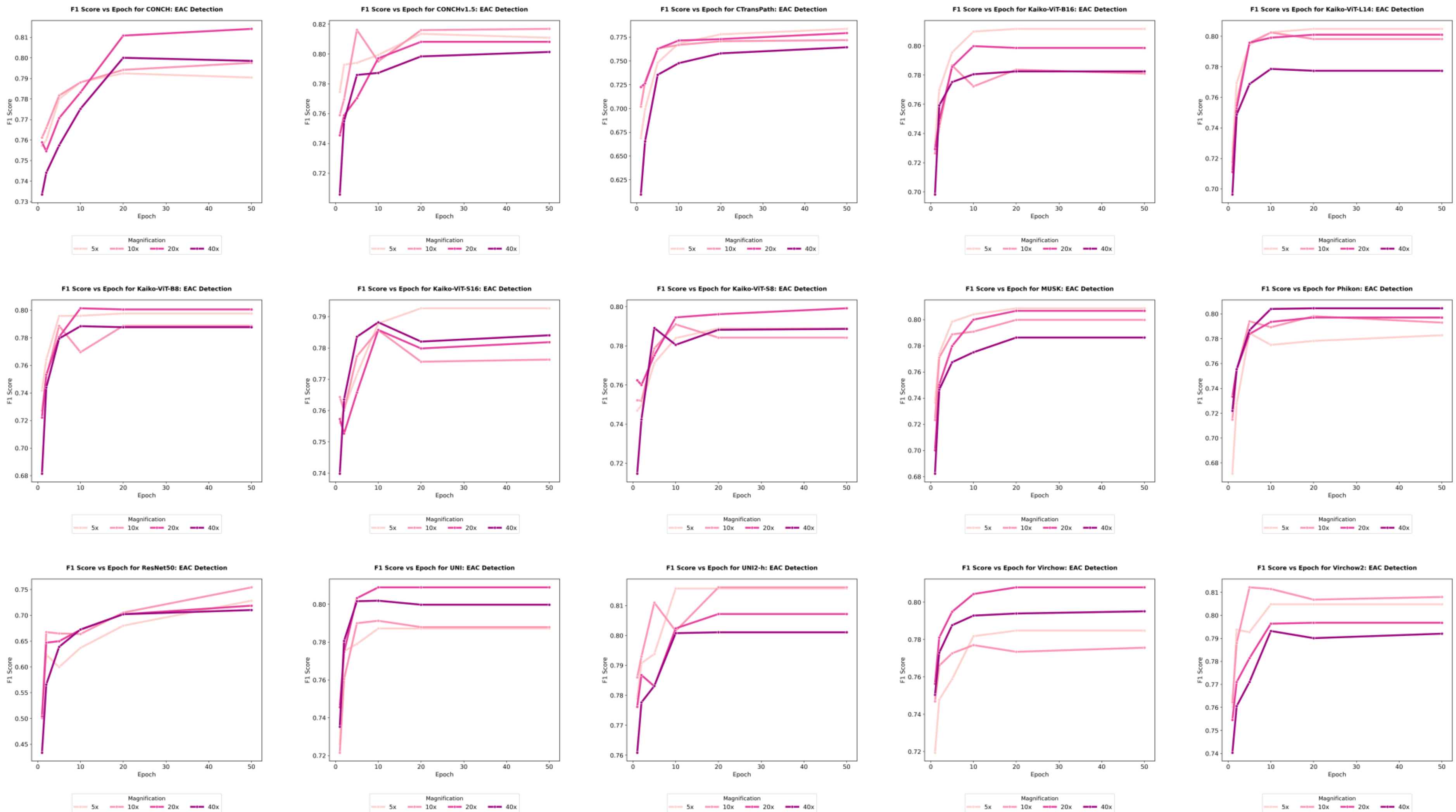


**Figure S7 | Models' F1 Score Trend for EAC Detection.**

**Table S2 | Micro and Weighted Performance Metrics of Models for EAC Detection.**

| Foundation Model | Magnification | F1-Score (Weighted Average)<br>Mean ± SD [95% CI Half-Width] | Precision (Micro Average)<br>Recall (Micro Average)<br>Recall (Weighted Average)<br>F1-Score (Micro Average)<br>Mean ± SD [95% CI Half-Width] | Precision (Weighted Average)<br>Mean ± SD [95% CI Half-Width] | Specificity (Weighted Average)<br>Mean ± SD [95% CI Half-Width] |
|---|---|---|---|---|---|
| Virchow2 | 40x | 0.925 ± 0.014 [± 0.010] | 0.913 ± 0.021 [± 0.015] | 0.947 ± 0.010 [± 0.007] | 0.841 ± 0.155 [± 0.111] |
| Virchow | 40x | 0.943 ± 0.019 [± 0.013] | 0.937 ± 0.023 [± 0.016] | 0.953 ± 0.014 [± 0.010] | 0.810 ± 0.110 [± 0.078] |
| UNI2-h | 40x | 0.942 ± 0.014 [± 0.010] | 0.936 ± 0.017 [± 0.012] | 0.952 ± 0.013 [± 0.009] | 0.813 ± 0.115 [± 0.083] |
| UNI | 40x | 0.940 ± 0.015 [± 0.011] | 0.932 ± 0.021 [± 0.015] | 0.953 ± 0.010 [± 0.007] | 0.853 ± 0.082 [± 0.058] |
| ResNet50 | 10x | 0.916 ± 0.015 [± 0.011] | 0.904 ± 0.019 [± 0.013] | 0.935 ± 0.016 [± 0.011] | 0.801 ± 0.080 [± 0.057] |
| Phikon-v2 | 20x | 0.950 ± 0.010 [± 0.007] | 0.947 ± 0.011 [± 0.008] | 0.956 ± 0.013 [± 0.009] | 0.767 ± 0.192 [± 0.138] |
| Phikon | 40x | 0.944 ± 0.014 [± 0.010] | 0.939 ± 0.017 [± 0.012] | 0.952 ± 0.014 [± 0.010] | 0.787 ± 0.128 [± 0.092] |
| MUSK | 20x | 0.940 ± 0.016 [± 0.012] | 0.933 ± 0.021 [± 0.015] | 0.952 ± 0.013 [± 0.009] | 0.836 ± 0.091 [± 0.065] |
| Lunit-ViT-S8 | 10x | 0.949 ± 0.018 [± 0.013] | 0.946 ± 0.020 [± 0.014] | 0.953 ± 0.016 [± 0.012] | 0.724 ± 0.138 [± 0.099] |
| Kaiko-ViT-S8 | 20x | 0.939 ± 0.015 [± 0.011] | 0.932 ± 0.018 [± 0.013] | 0.951 ± 0.013 [± 0.009] | 0.830 ± 0.110 [± 0.078] |
| Kaiko-ViT-S16 | 40x | 0.942 ± 0.014 [± 0.010] | 0.937 ± 0.017 [± 0.012] | 0.952 ± 0.013 [± 0.009] | 0.811 ± 0.110 [± 0.079] |
| Kaiko-ViT-L14 | 20x | 0.930 ± 0.015 [± 0.011] | 0.920 ± 0.021 [± 0.015] | 0.948 ± 0.010 [± 0.007] | 0.847 ± 0.089 [± 0.063] |
| Kaiko-ViT-B8 | 20x | 0.941 ± 0.016 [± 0.012] | 0.935 ± 0.020 [± 0.015] | 0.952 ± 0.013 [± 0.010] | 0.804 ± 0.123 [± 0.088] |
| Kaiko-ViT-B16 | 40x | 0.940 ± 0.015 [± 0.011] | 0.934 ± 0.019 [± 0.013] | 0.950 ± 0.013 [± 0.009] | 0.804 ± 0.094 [± 0.067] |
| H-Optimus-0 | 40x | 0.955 ± 0.009 [± 0.007] | 0.953 ± 0.010 [± 0.007] | 0.959 ± 0.009 [± 0.007] | 0.787 ± 0.097 [± 0.070] |
| Hibou-L | 40x | 0.946 ± 0.016 [± 0.012] | 0.941 ± 0.019 [± 0.014] | 0.954 ± 0.013 [± 0.010] | 0.798 ± 0.143 [± 0.102] |
| Prov-Gigapath | 10x | 0.949 ± 0.014 [± 0.010] | 0.947 ± 0.016 [± 0.011] | 0.953 ± 0.013 [± 0.009] | 0.738 ± 0.103 [± 0.073] |
| CTransPath | 5x | 0.937 ± 0.014 [± 0.010] | 0.930 ± 0.017 [± 0.012] | 0.949 ± 0.013 [± 0.009] | 0.820 ± 0.108 [± 0.077] |
| CONCHv1.5 | 20x | 0.935 ± 0.024 [± 0.017] | 0.926 ± 0.033 [± 0.023] | 0.950 ± 0.015 [± 0.010] | 0.829 ± 0.102 [± 0.073] |
| CONCH | 40x | 0.939 ± 0.013 [± 0.009] | 0.932 ± 0.016 [± 0.011] | 0.951 ± 0.011 [± 0.008] | 0.839 ± 0.067 [± 0.048] |

SD: Standard Deviation; CI: Confidence Interval.

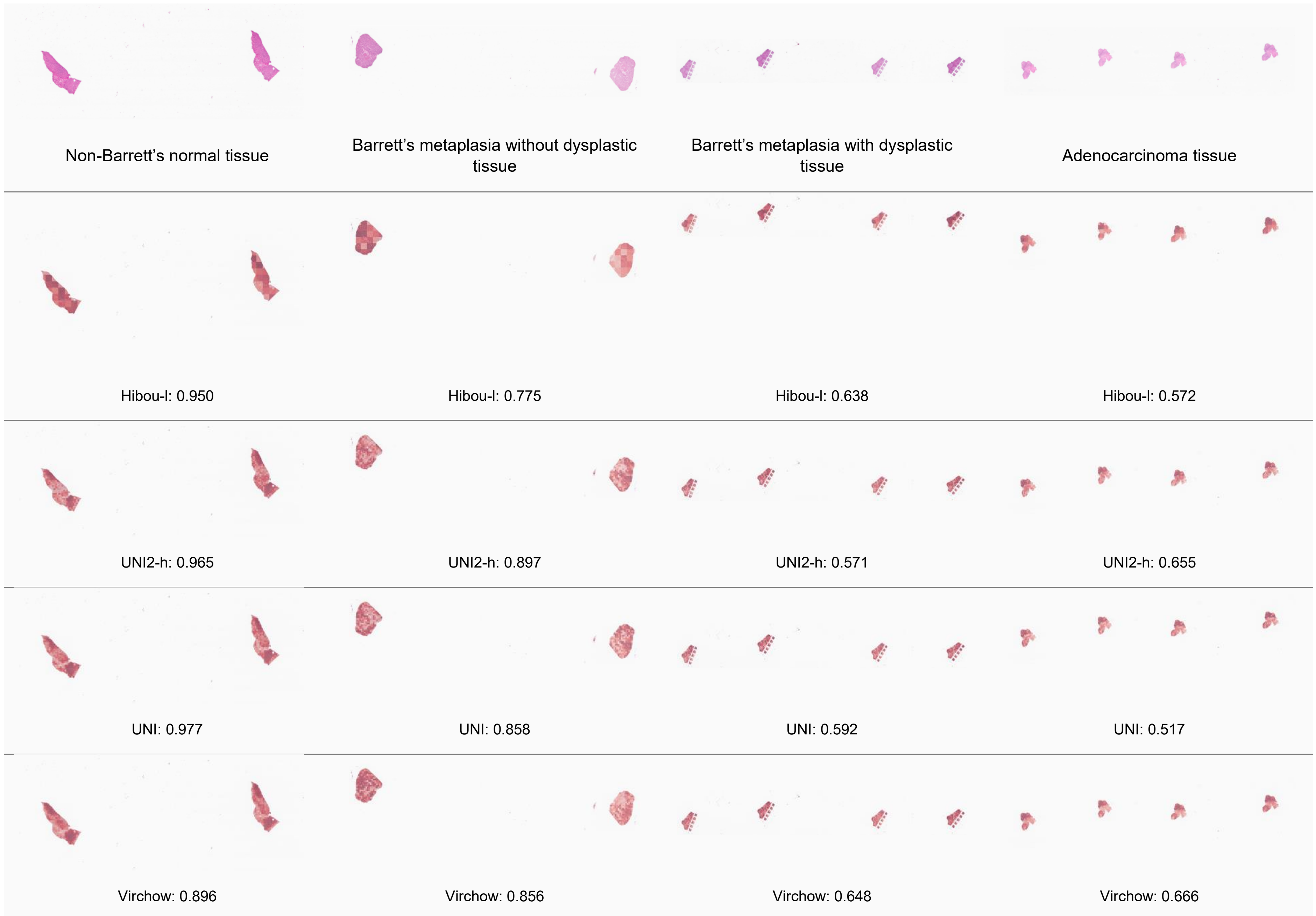
Non-Barrett's normal tissue
Barrett's metaplasia without dysplastic tissue
Barrett's metaplasia with dysplastic tissue
Adenocarcinoma tissue
Hibou-l: 0.950
Hibou-l: 0.775
Hibou-l: 0.638
Hibou-l: 0.572
UNI2-h: 0.965
UNI2-h: 0.897
UNI2-h: 0.571
UNI2-h: 0.655
UNI: 0.977
UNI: 0.858
UNI: 0.592
UNI: 0.517
Virchow: 0.896
Virchow: 0.856
Virchow: 0.648
Virchow: 0.666

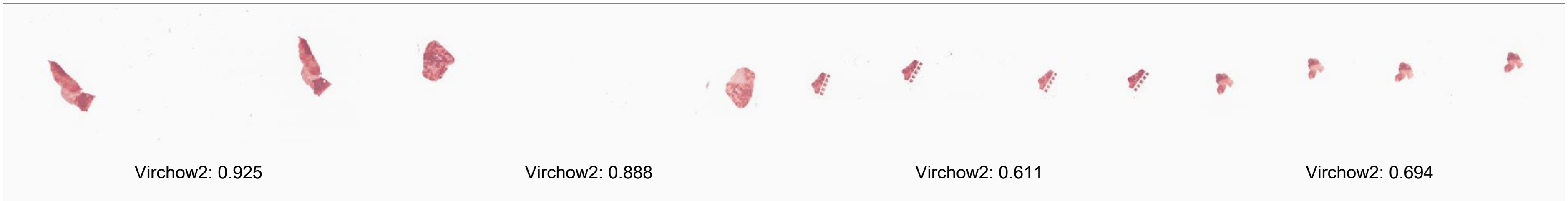


**Figure S8 | Pathologist Validation of Attention Heatmaps.** Attention maps for four randomly sampled whole slide images (WSIs), which correctly predicted each from one of the morphological subtypes, were generated. Heatmaps derived from the top five expert-consistent (ExCo) models for morphological subtyping, with each attention heatmap corresponding to one model. Thumbnails of the original WSIs and the heatmaps are shown.

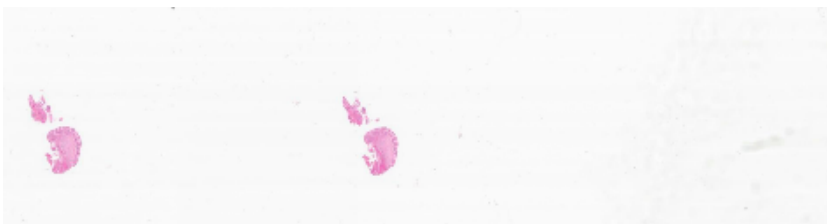
Barrett's metaplasia with dysplastic tissue

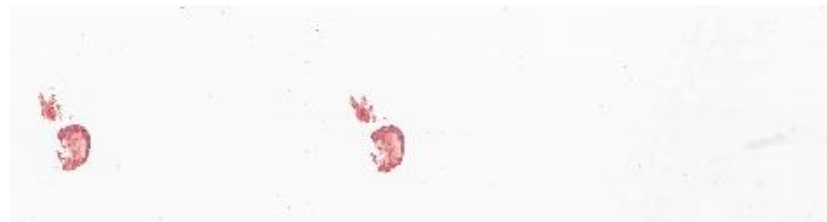
Virchow2 (Incorrect: Barrett's metaplasia without dysplastic tissue)

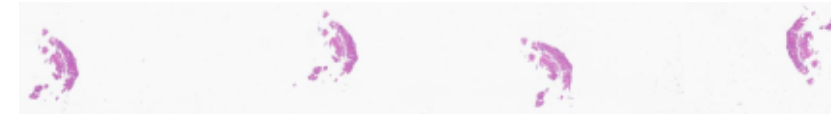
Barrett's metaplasia with dysplastic tissue

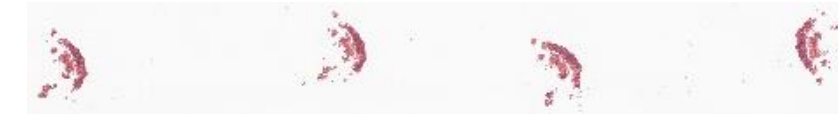
Virchow2 (Correct: Barrett's metaplasia with dysplastic tissue)

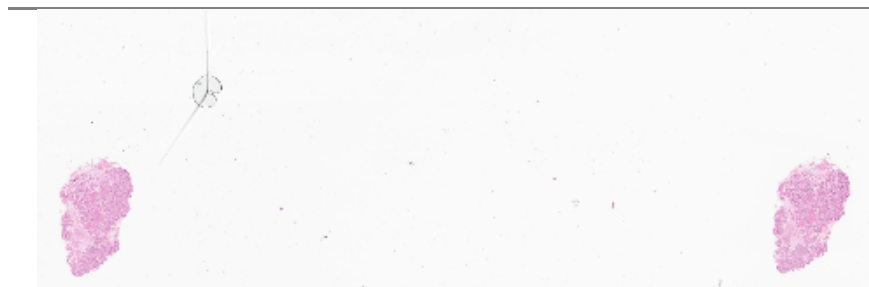
Barrett's metaplasia with dysplastic tissue

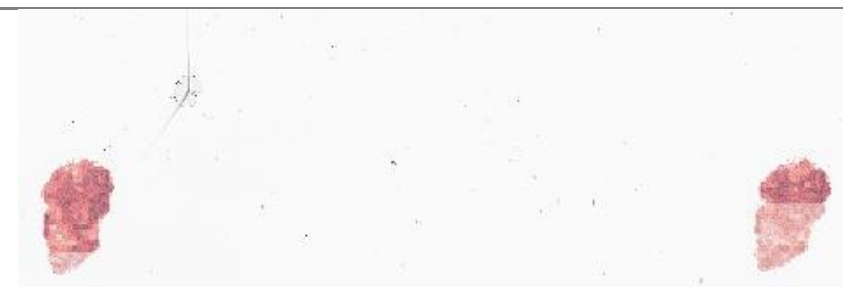
Virchow2 (Incorrect: Barrett's metaplasia without dysplastic tissue)

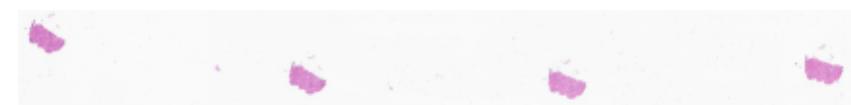
Barrett's metaplasia with dysplastic tissue

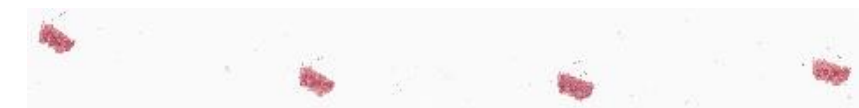
Virchow2 (Incorrect: Barrett's metaplasia without dysplastic tissue)

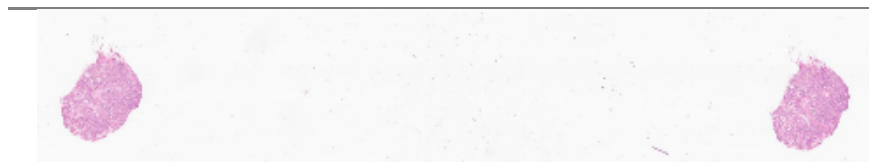
Barrett's metaplasia with dysplastic tissue

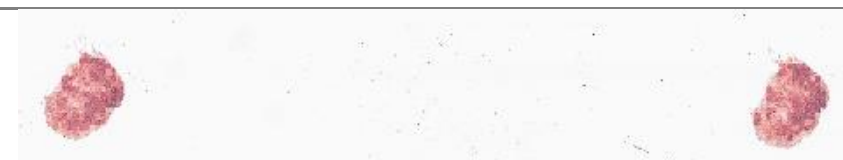
Virchow2 (Correct: Barrett's metaplasia with dysplastic tissue)

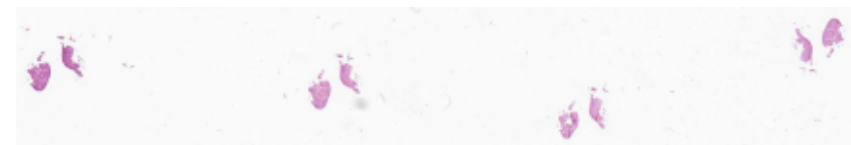
Barrett's metaplasia with dysplastic tissue

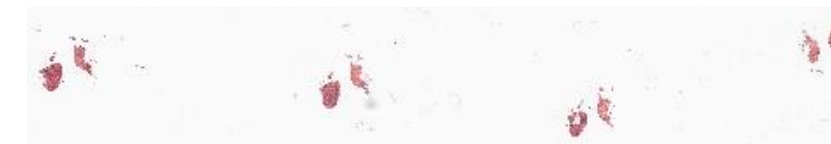
Virchow2(Correct: Barrett's metaplasia with dysplastic tissue)

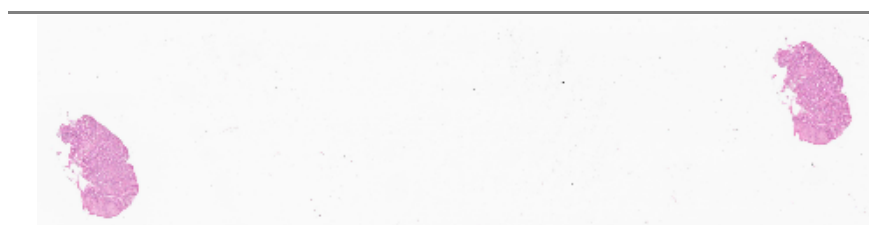
Barrett's metaplasia with dysplastic tissue

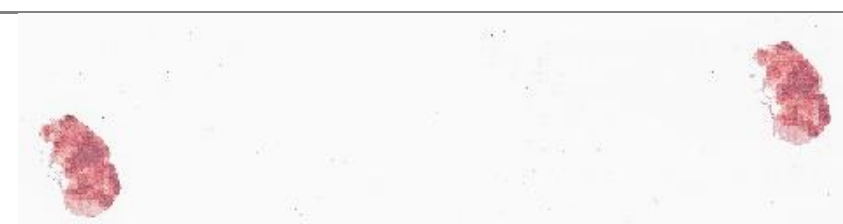
Virchow2 (Incorrect: Barrett's metaplasia without dysplastic tissue)

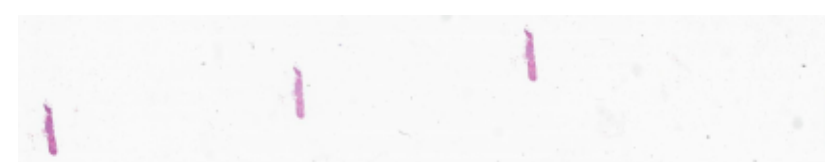
Adenocarcinoma

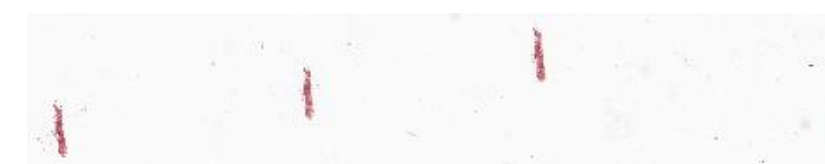
Virchow2 (Correct: Adenocarcinoma)

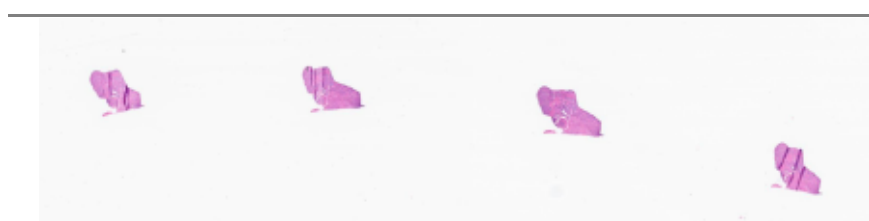
Barrett's metaplasia with dysplastic tissue

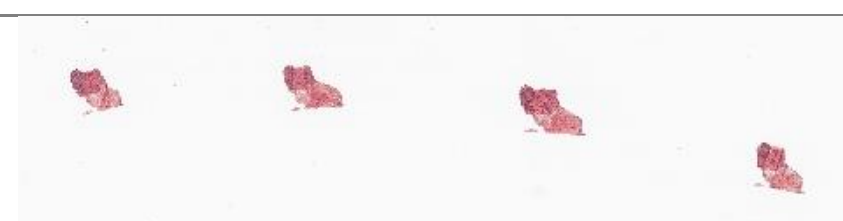
Virchow2 (Incorrect: Adenocarcinoma)

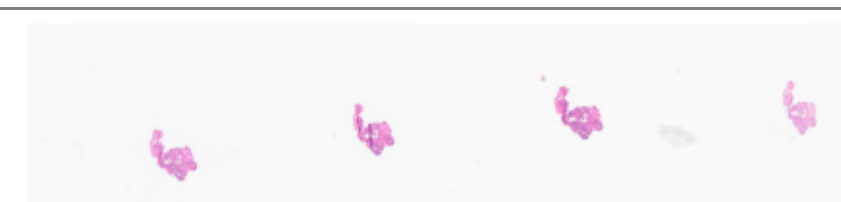
Barrett's metaplasia with dysplastic tissue

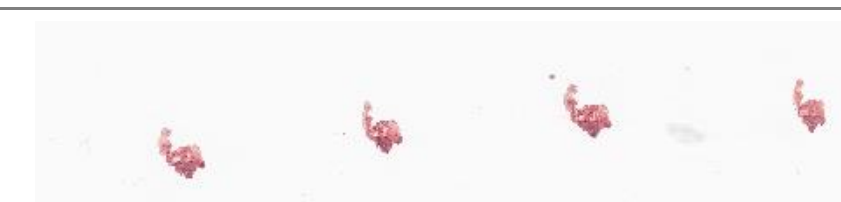
Virchow2 (Incorrect: Adenocarcinoma)

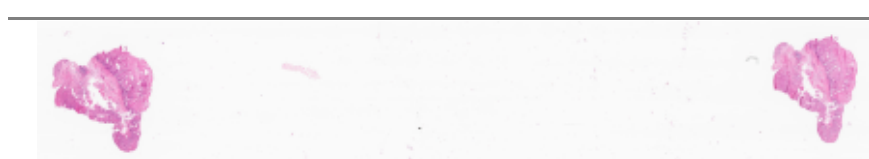
Barrett's metaplasia with dysplastic tissue

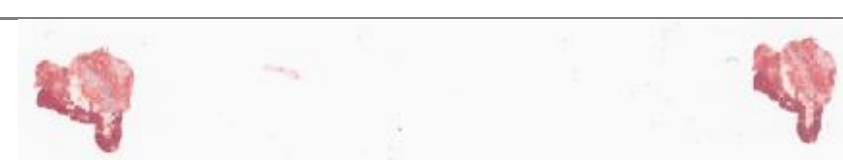
UNI2-h (Correct: Barrett's metaplasia with dysplastic tissue)

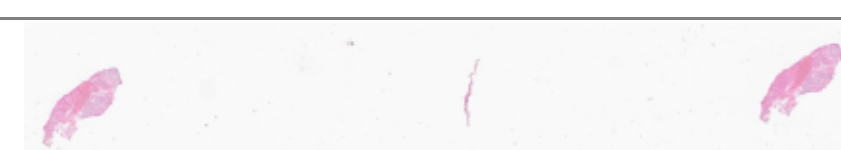
Adenocarcinoma

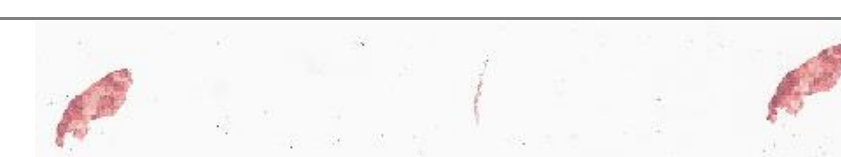
UNI2-h (Incorrect: Barrett's metaplasia with dysplastic tissue)

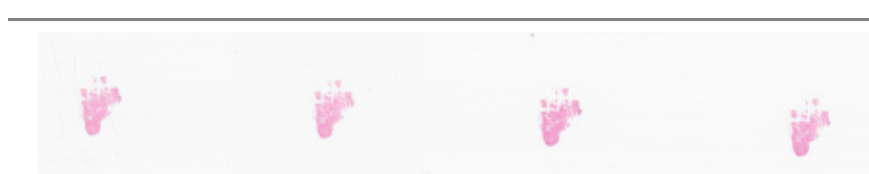
Barrett's metaplasia with dysplastic tissue

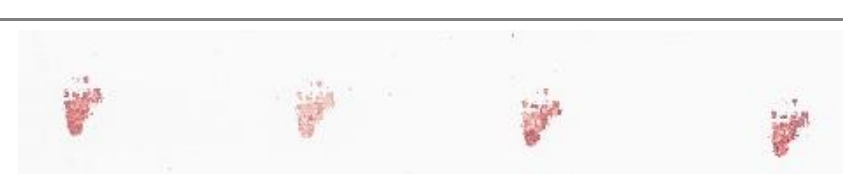
UNI2-h (Incorrect: Barrett's metaplasia without dysplastic tissue)

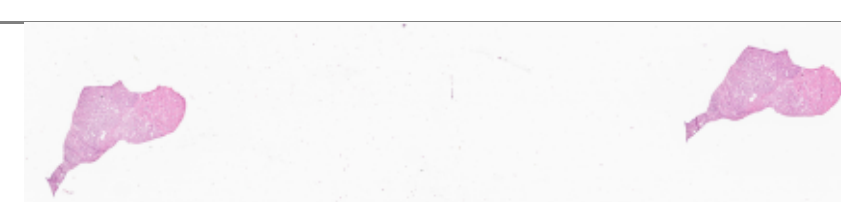
Adenocarcinoma

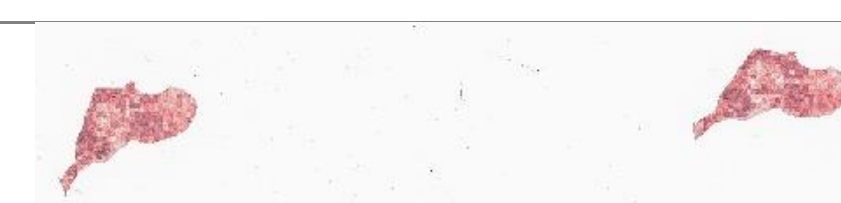
UNI2-h (Incorrect: Barrett's metaplasia with dysplastic tissue)

| | | | |
|---|---|---|---|
| 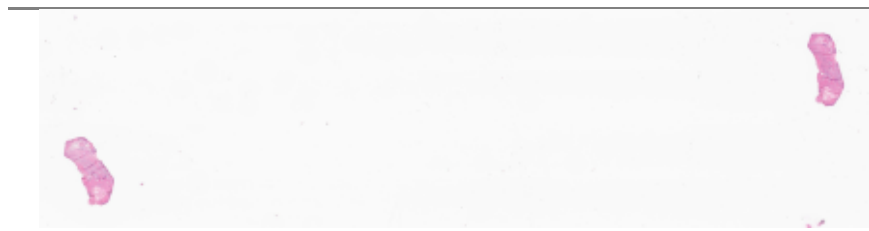 | 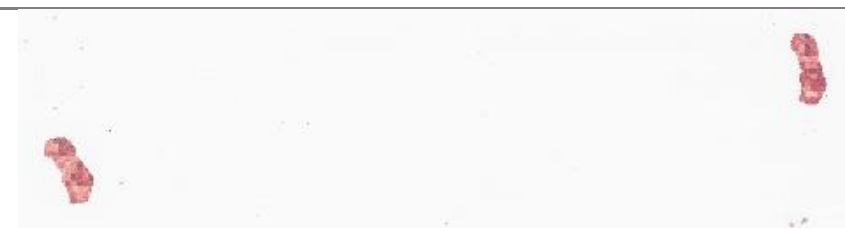 | 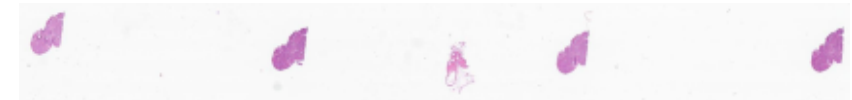 | 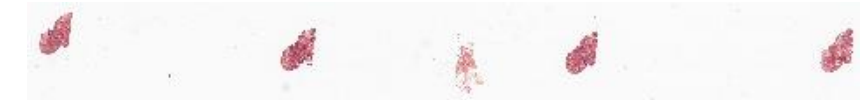 |
| Adenocarcinoma | UNI2-h (Correct: Adenocarcinoma) | Barrett's metaplasia with dysplastic tissue | UNI2-h (Correct: Barrett's metaplasia with dysplastic tissue) |
| 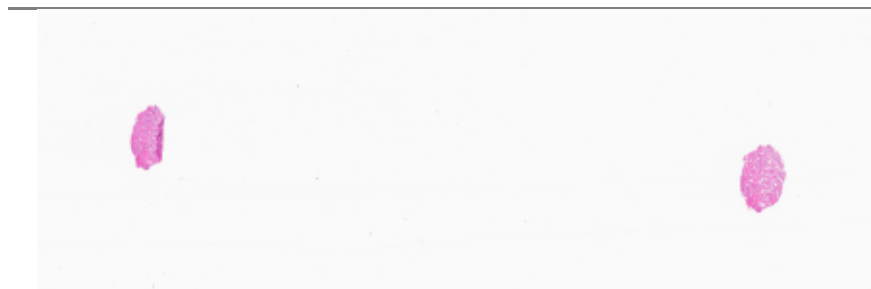 | 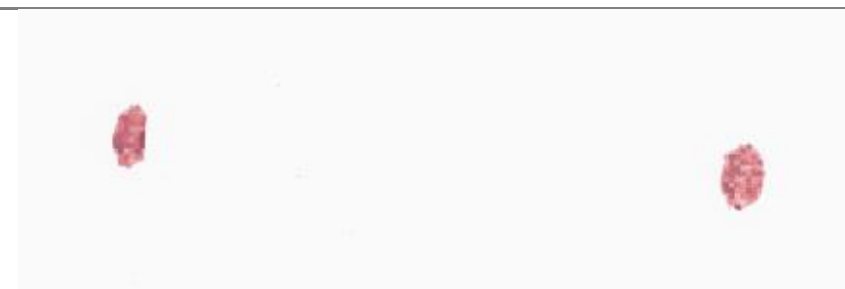 | 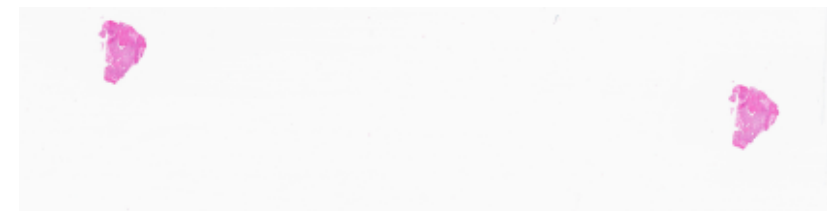 | 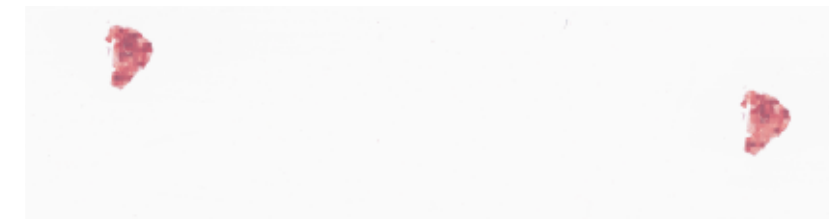 |
| Barrett's metaplasia with dysplastic tissue | Virchow (Correct: Barrett's metaplasia with dysplastic tissue) | Barrett's metaplasia with dysplastic tissue | Virchow (Correct: Barrett's metaplasia with dysplastic tissue) |
| 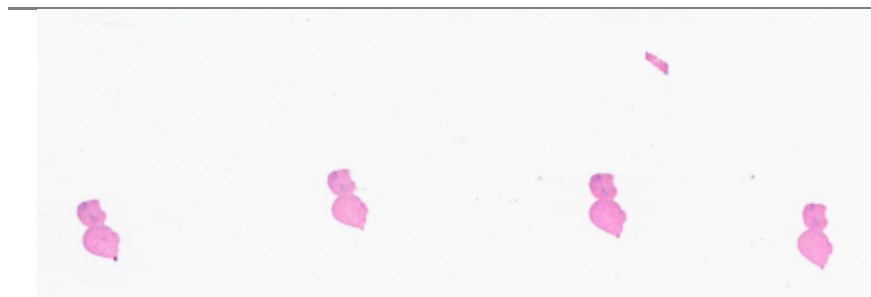 | 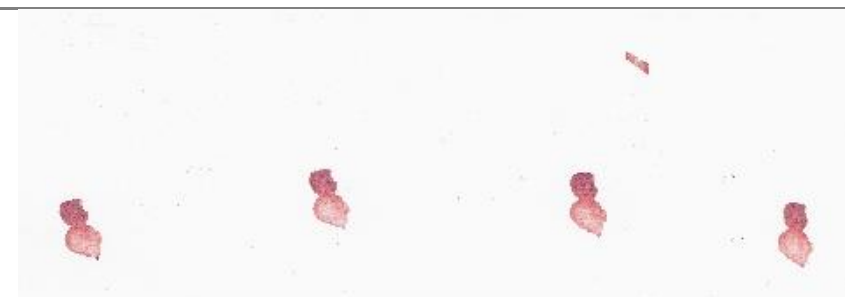 | 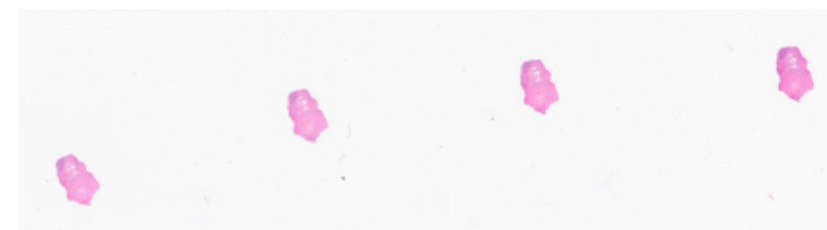 | 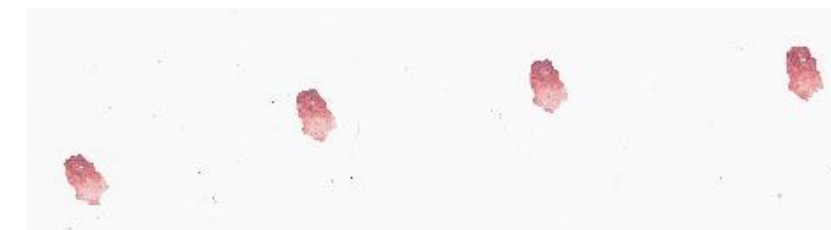 |
| Barrett's metaplasia with dysplastic tissue | Virchow (Incorrect: Adenocarcinoma) | Barrett's metaplasia with dysplastic tissue | Virchow (Incorrect: Adenocarcinoma) |
| 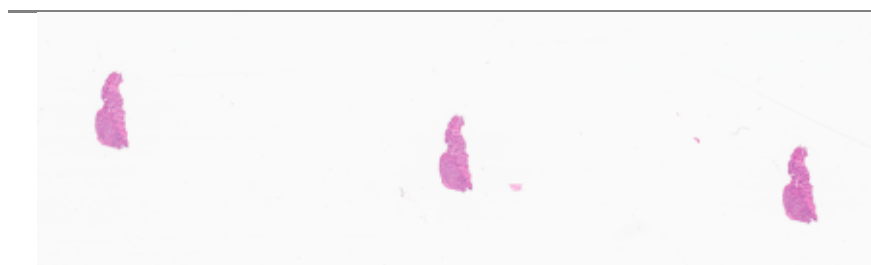 | 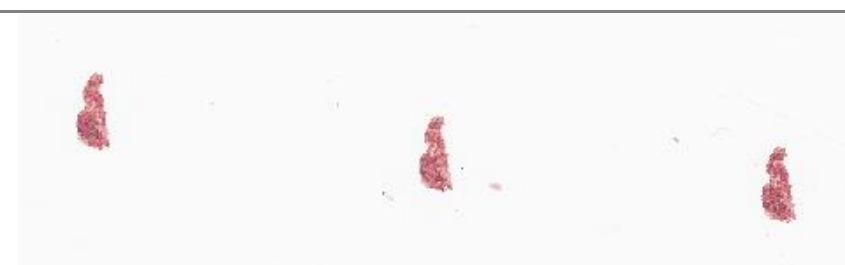 | 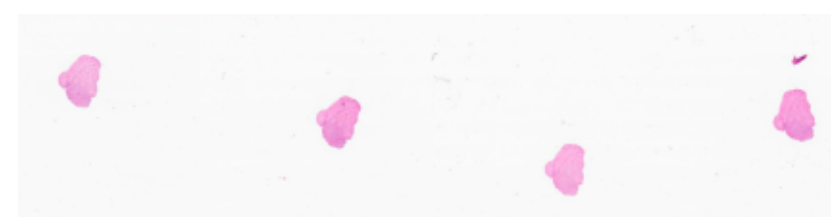 | 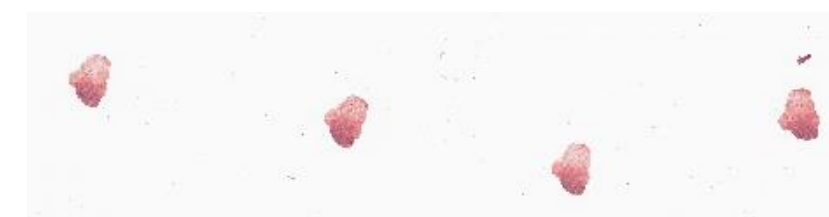 |
| Barrett's metaplasia with dysplastic tissue | Virchow (Incorrect: Barrett's metaplasia without dysplastic tissue) | Barrett's metaplasia with dysplastic tissue | Virchow (Correct: Barrett's metaplasia with dysplastic tissue |
| 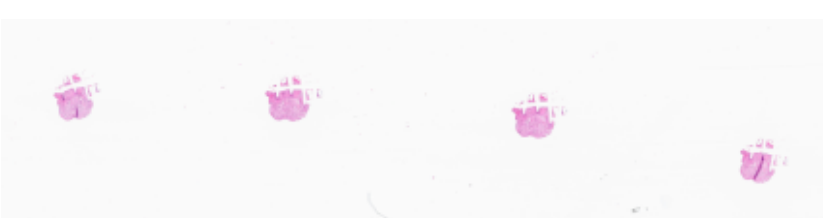 | 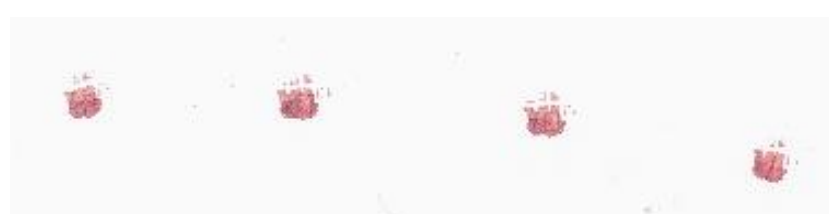 | 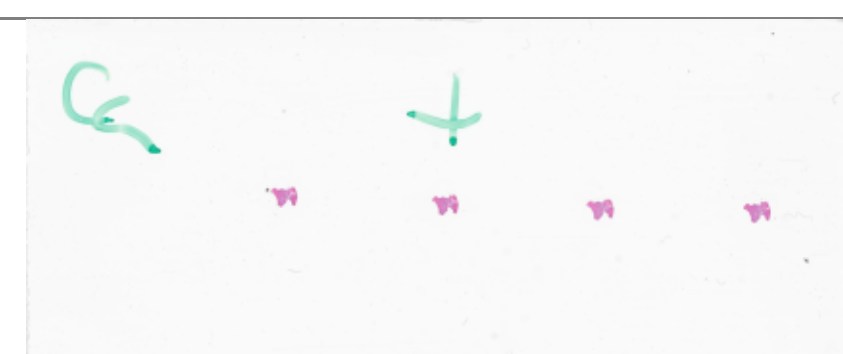 | 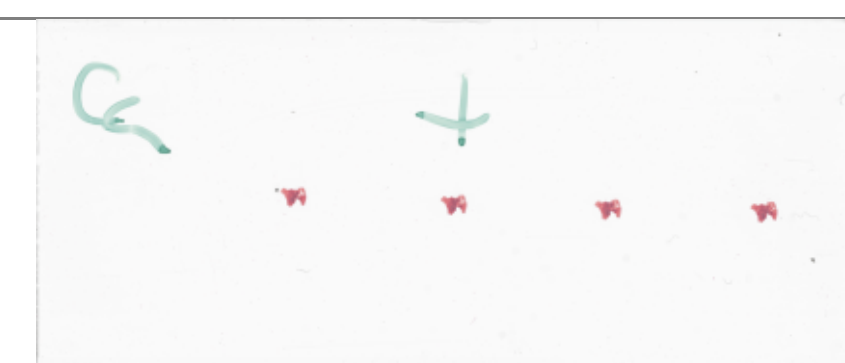 |
| Barrett's metaplasia with dysplastic tissue | Virchow (Incorrect: Adenocarcinoma) | Barrett's metaplasia with dysplastic tissue | Virchow (Incorrect: Adenocarcinoma) |
| 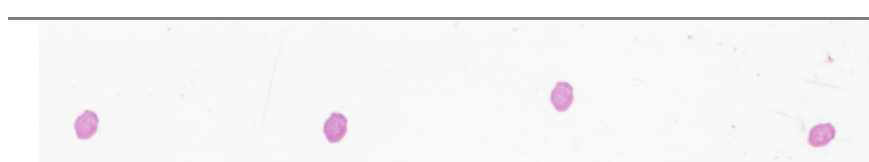 | 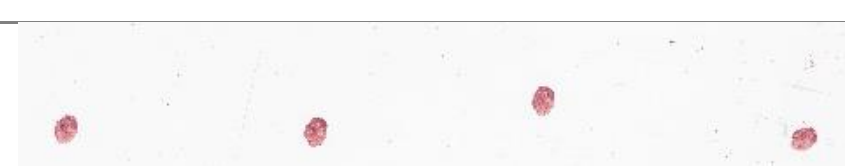 | 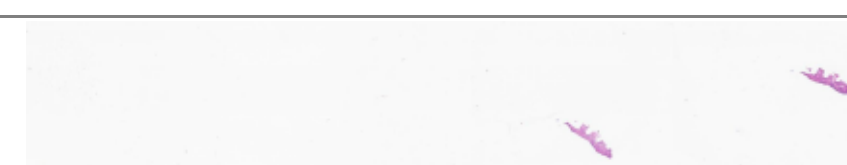 | 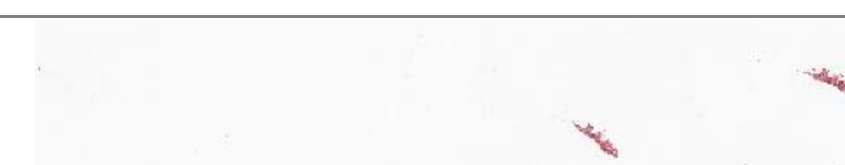 |
| Adenocarcinoma | Virchow (Incorrect: Barrett's metaplasia with dysplastic tissue) | Barrett's metaplasia with dysplastic tissue | UNI (Correct: Barrett's metaplasia with dysplastic tissue) |

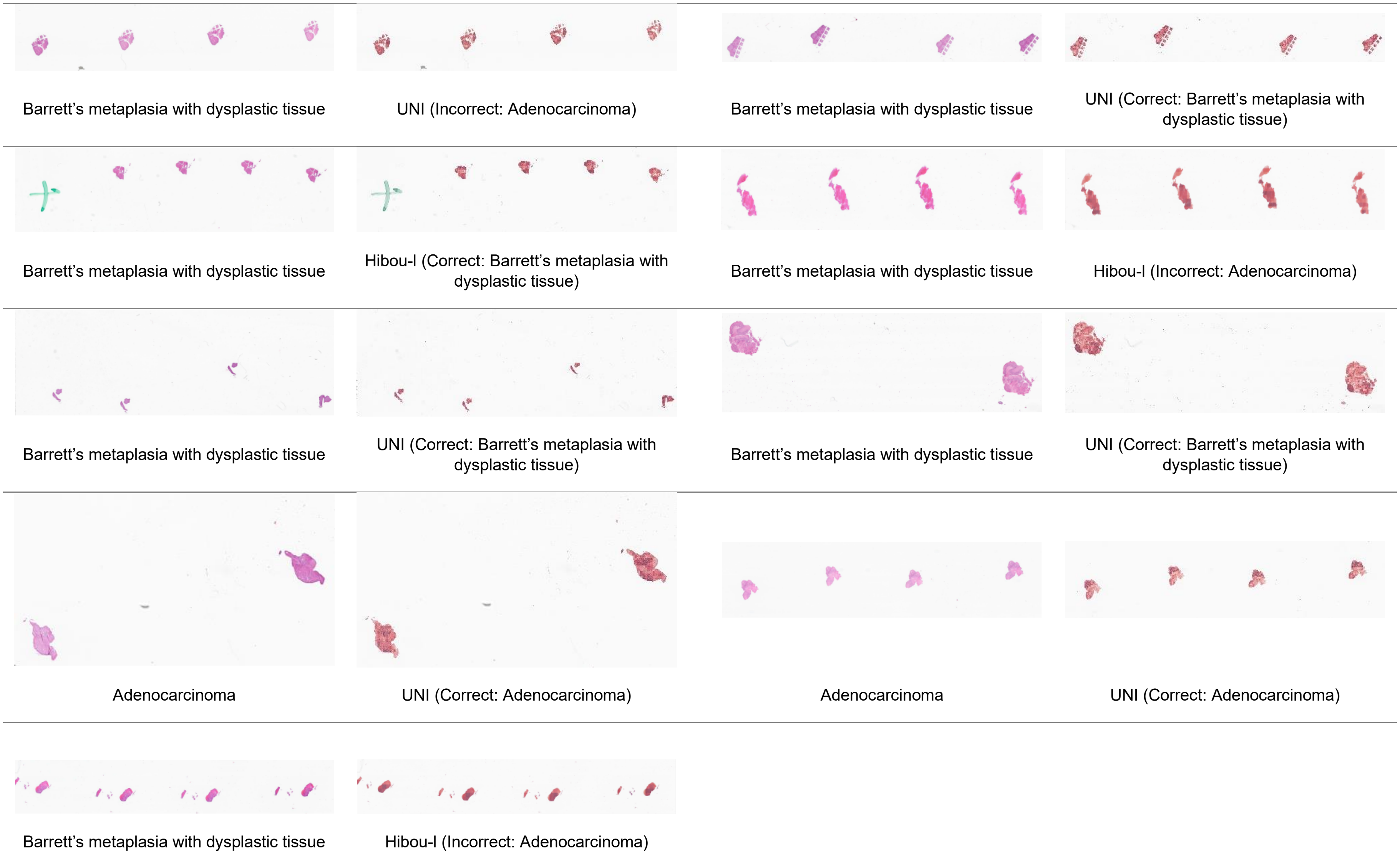


**Figure S9 | Attention Heatmap Validation for Diagnostic Tissue Regions.** Attention maps for 35 randomly sampled whole slide images (WSIs) from dysplasia and adenocarcinoma were generated, with each attention heatmap derived from the morphological subtyping predictions of the top five expert-consistent (ExCo) models. Thumbnails of the original WSIs and the heatmaps are shown.
