# Supplementary material for: Towards reliable morphological subtyping and cancer detection on Barrett’s esophagus pathology through ensembling foundation models
Source: NPJ Digit Med. 2026 Oct 21;9:811. doi: 10.1038/s41746-026-03329-3 (PMC13694699; doi:10.1038/s41746-026-03329-3)
Supplement: Supplementary file 2 [file 41746_2026_3329_MOESM2_ESM.pdf]

nature portfolio

Corresponding author(s): Azar Kazemi
Last updated by author(s): 24.06.2026

Reporting Summary

Nature Portfolio wishes to improve the reproducibility of the work that we publish. This form provides structure for consistency and transparency in reporting. For further information on Nature Portfolio policies, see our Editorial Policies and the Editorial Policy Checklist.

Please do not complete any field with "not applicable" or n/a. Refer to the help text for what text to use if an item is not relevant to your study.
For final submission: please carefully check your responses for accuracy; you will not be able to make changes later.

Statistics

For all statistical analyses, confirm that the following items are present in the figure legend, table legend, main text, or Methods section.

n/a	Confirmed	
☐	☑	The exact sample size (*n*) for each experimental group/condition, given as a discrete number and unit of measurement
☐	☑	A statement on whether measurements were taken from distinct samples or whether the same sample was measured repeatedly
☐	☑	The statistical test(s) used AND whether they are one- or two-sided<br>*Only common tests should be described solely by name; describe more complex techniques in the Methods section.*
☑	☐	A description of all covariates tested
☐	☑	A description of any assumptions or corrections, such as tests of normality and adjustment for multiple comparisons
☐	☑	A full description of the statistical parameters including central tendency (e.g. means) or other basic estimates (e.g. regression coefficient) AND variation (e.g. standard deviation) or associated estimates of uncertainty (e.g. confidence intervals)
☐	☑	For null hypothesis testing, the test statistic (e.g. *F*, *t*, *r*) with confidence intervals, effect sizes, degrees of freedom and *P* value noted<br>*Give P values as exact values whenever suitable.*
☑	☐	For Bayesian analysis, information on the choice of priors and Markov chain Monte Carlo settings
☐	☑	For hierarchical and complex designs, identification of the appropriate level for tests and full reporting of outcomes
☑	☐	Estimates of effect sizes (e.g. Cohen's *d*, Pearson's *r*), indicating how they were calculated

Our web collection on statistics for biologists contains articles on many of the points above.

Software and code

Policy information about availability of computer code

Data collection	Leica Aperio GT 450 DX scanners, data was exported from BarrettNet registry
Data analysis	Modeling and experiments rely on python (version 3.11.7), the OpenSlide package (version 1.3.1), pytorch package (2.5.1+cu124), TRIDENT framework and CLAM repository.<br>Custome code was generated for the evaluation.

For manuscripts utilizing custom algorithms or software that are central to the research but not yet described in published literature, software must be made available to editors and reviewers. We strongly encourage code deposition in a community repository (e.g. GitHub). See the Nature Portfolio guidelines for submitting code & software for further information.

Data

Policy information about availability of data

All manuscripts must include a data availability statement. This statement should provide the following information, where applicable:

- Accession codes, unique identifiers, or web links for publicly available datasets
- A description of any restrictions on data availability
- For clinical datasets or third party data, please ensure that the statement adheres to our policy

Pathology slides, digital pathology and associated clinical data are not publicly available due to legal and patient privacy consideration and institutional data-sharing restriction.

Research involving human participants, their data, or biological material

Policy information about studies with human participants or human data. See also policy information about sex, gender (identity/presentation), and sexual orientation and race, ethnicity and racism.

Reporting on sex and gender	Dataset which was used in this study was not derived from the cohort study on the basis of sex and gender. This information was not included in the analysis.
Reporting on race, ethnicity, or other socially relevant groupings	The dataset have been used in this study was not constructed on the basis of race, ethnicity, or other socially relevant demographic variables and therefore were not included in the analysis.
Population characteristics	The available population characteristics were limited to diagnostic categories derived from histopathological assessment. Demographic variables such as sex, age, gender, race and ethnicity were notused in the investigations.
Recruitment	A retrospective multicenter cohort of Barrett esophagus biopsies collected from participating centers within BarrettNet.
Ethics oversight	This study was conducted in accordance with the Declaration of Helsinki and approved by the Technical University of Munich Ethics Committee (approval no. 2023-665-S-CB). The need for informed consent was waived because all data were collected as part of the BarrettNet study (Wiethaler, M. et al., 2019) earlier and de-identified for this study.

Note that full information on the approval of the study protocol must also be provided in the manuscript.

Field-specific reporting

Please select the one below that is the best fit for your research. If you are not sure, read the appropriate sections before making your selection.

☑ Life sciences ☐ Behavioural & social sciences ☐ Ecological, evolutionary & environmental sciences

For a reference copy of the document with all sections, see nature.com/documents/nr-reporting-summary-flat.pdf

Life sciences study design

All studies must disclose on these points even when the disclosure is negative.

Sample size	For the benchmark for both tasks, sample size was chosen based on availability of target data and digital images.
Data exclusions	Slides were excluded if they were not H&E stained and due to missing values in their metadata (identifier, barcode or associated clinical data), were not retrivable or usable.
Replication	Perfrmance was evaluated across 10 Monte Carlo cross-validation iterations, and results are reported as mean ± SD and/or 95% CI across those iterations
Randomization	Random assignment of slides to each partition within each Monte Carlo cross-validation iterations was done with patient-level stratification to prevent data leakage between sets.
Blinding	No blinding was applied during model training and evaluation because of weakly supervised learning required access to the diagnostic lables. For pathologist validation of models' outputs as attention heatmaps, pathologists were not blinded to the diagnosis but they were blinded to the predictions, perfromance of models and the name of models.

Behavioural & social sciences study design

All studies must disclose on these points even when the disclosure is negative.

Study description	No behavioral intervention was perfromed.
Research sample	
Sampling strategy	
Data collection	
Timing	
Data exclusions	
Non-participation	
Randomization	

Ecological, evolutionary & environmental sciences study design

All studies must disclose on these points even when the disclosure is negative.

Study description	Not applicable.
Research sample	
Sampling strategy	
Data collection	
Timing and spatial scale	
Data exclusions	
Reproducibility	
Randomization	
Blinding	

Did the study involve field work? ☐ Yes ☑ No

Field work, collection and transport

Field conditions	Not applicable.
Location	
Access & import/export	
Disturbance	

Reporting for specific materials, systems and methods

We require information from authors about some types of materials, experimental systems and methods used in many studies. Here, indicate whether each material, system or method listed is relevant to your study. If you are not sure if a list item applies to your research, read the appropriate section before selecting a response.

Materials & experimental systems

n/a	Involved in the study	
☑	☐	Antibodies
☑	☐	Eukaryotic cell lines
☑	☐	Palaeontology and archaeology
☑	☐	Animals and other organisms
☑	☐	Clinical data
☑	☐	Dual use research of concern
☑	☐	Plants

Methods

n/a	Involved in the study	
☑	☐	ChIP-seq
☑	☐	Flow cytometry
☑	☐	MRI-based neuroimaging

Antibodies

Antibodies used	Not applicable.
Validation	

Eukaryotic cell lines

Policy information about cell lines and Sex and Gender in Research

Cell line source(s)	Not applicable.
Authentication	
Mycoplasma contamination	
Commonly misidentified lines (See ICLAC register)	

Palaeontology and Archaeology

Specimen provenance	Not applicable.
Specimen deposition	
Dating methods	

☐ Tick this box to confirm that the raw and calibrated dates are available in the paper or in Supplementary Information.

Ethics oversight	

Note that full information on the approval of the study protocol must also be provided in the manuscript.

Animals and other research organisms

Policy information about studies involving animals; ARRIVE guidelines recommended for reporting animal research, and Sex and Gender in Research

Laboratory animals	Not applicable.
Wild animals	
Reporting on sex	
Field-collected samples	
Ethics oversight	

Note that full information on the approval of the study protocol must also be provided in the manuscript.

Clinical data

Policy information about clinical studies

All manuscripts should comply with the ICMJE guidelines for publication of clinical research and a completed CONSORT checklist must be included with all submissions.

Clinical trial registration	Not applicable.
Study protocol	
Data collection	
Outcomes	

Dual use research of concern

Policy information about dual use research of concern

Hazards

Could the accidental, deliberate or reckless misuse of agents or technologies generated in the work, or the application of information presented in the manuscript, pose a threat to:

No | Yes

- [x] [ ] Public health
- [x] [ ] National security
- [x] [ ] Crops and/or livestock
- [x] [ ] Ecosystems
- [x] [ ] Any other significant area

Experiments of concern

Does the work involve any of these experiments of concern:

No | Yes

- [x] [ ] Demonstrate how to render a vaccine ineffective
- [x] [ ] Confer resistance to therapeutically useful antibiotics or antiviral agents
- [x] [ ] Enhance the virulence of a pathogen or render a nonpathogen virulent
- [x] [ ] Increase transmissibility of a pathogen
- [x] [ ] Alter the host range of a pathogen
- [x] [ ] Enable evasion of diagnostic/detection modalities
- [x] [ ] Enable the weaponization of a biological agent or toxin
- [x] [ ] Any other potentially harmful combination of experiments and agents

Plants

Seed stocks	Not applicable.
Novel plant genotypes	
Authentication	

ChIP-seq

Data deposition

- [ ] Confirm that both raw and final processed data have been deposited in a public database such as GEO.
- [ ] Confirm that you have deposited or provided access to graph files (e.g. BED files) for the called peaks.

Data access links<br>*May remain private before publication.*	Not applicable.
Files in database submission	
Genome browser session<br>(e.g. UCSC)	

Methodology

Replicates	
Sequencing depth	
Antibodies	
Peak calling parameters	
Data quality	

Software

Flow Cytometry

Plots

Confirm that:

- [ ] The axis labels state the marker and fluorochrome used (e.g. CD4-FITC).
- [ ] The axis scales are clearly visible. Include numbers along axes only for bottom left plot of group (a 'group' is an analysis of identical markers).
- [ ] All plots are contour plots with outliers or pseudocolor plots.
- [ ] A numerical value for number of cells or percentage (with statistics) is provided.

Methodology

Sample preparation	Not applicable.
Instrument	
Software	
Cell population abundance	
Gating strategy	

- [ ] Tick this box to confirm that a figure exemplifying the gating strategy is provided in the Supplementary Information.

Magnetic resonance imaging

Experimental design

Design type	Not applicable.
Design specifications	
Behavioral performance measures	
Imaging type(s)	
Field strength	
Sequence & imaging parameters	
Area of acquisition	

Diffusion MRI [ ] Used [ ] Not used

Preprocessing

Preprocessing software	Not applicable.
Normalization	
Normalization template	
Noise and artifact removal	
Volume censoring	

Statistical modeling & inference

Model type and settings	
Effect(s) tested	

Specify type of analysis: ☐ Whole brain ☐ ROI-based ☐ Both

Statistic type for inference

(See Eklund et al. 2016)

Correction

Models & analysis

n/a	Involved in the study
☐	☐ Functional and/or effective connectivity
☐	☐ Graph analysis
☐	☐ Multivariate modeling or predictive analysis

Functional and/or effective connectivity

Graph analysis

Multivariate modeling and predictive analysis

This checklist template is licensed under a Creative Commons Attribution 4.0 International License, which permits use, sharing, adaptation, distribution and reproduction in any medium or format, as long as you give appropriate credit to the original author(s) and the source, provide a link to the Creative Commons license, and indicate if changes were made. The images or other third party material in this article are included in the article's Creative Commons license, unless indicated otherwise in a credit line to the material. If material is not included in the article's Creative Commons license and your intended use is not permitted by statutory regulation or exceeds the permitted use, you will need to obtain permission directly from the copyright holder. To view a copy of this license, visit http://creativecommons.org/licenses/by/4.0/
